# Accelerated Molecular Transportation in the Brain Extracellular Space with 755-nm Light Attenuates Post-Stroke Cognitive Impairment in Rats

**DOI:** 10.34133/cbsystems.0262

**Published:** 2025-05-06

**Authors:** Liu Yang, Yajuan Gao, Leonor Serrano Lopes, Jingge Lian, Wanyi Fu, Hanbo Tan, Shuangfeng Yang, Zhaoheng Xie, Yixing Huang, Jicong Zhang, Yanye Lu, Hao Tang, Bo Xiong, Xunbin Wei, Lide Xie, Yun Peng, Xinyu Liu, Hongbin Han

**Affiliations:** ^1^ Department of Radiology, Peking University Third Hospital, Beijing 100191, China.; ^2^ Beijing Key Laboratory of Magnetic Resonance Imaging Technology, Beijing 100191, China.; ^3^ Department of Nuclear Medicine, Inselspital, Bern University Hospital, University of Bern, Bern 3010, Switzerland.; ^4^ Graduate School for Cellular and Biomedical Sciences, University of Bern, Bern 3012, Switzerland.; ^5^ Institute of Medical Technology, Peking University Health Science Center, Beijing 100191, China.; ^6^ Department of Electronic Engineering, Tsinghua University, Beijing 100084, China.; ^7^ Department of Radiology, Beijing Children’s Hospital, Capital Medical University, National Center for Children’ Health, Beijing 100045, China.; ^8^ School of Biological Science and Medical Engineering, Beihang University, Beijing 100191, China.; ^9^ School of Computer Science, Peking University, Beijing 100871, China.; ^10^ Chengde Medical University, Chengde, Hebei 067000, China.

## Abstract

Ischemic stroke exacts a heavy toll in death and disability worldwide. After ischemic stroke, the accumulation of pathobiomolecules in the brain extracellular space (ECS) will exacerbate neurological damage and cognitive impairment. Photobiomodulation (PBM) has been demonstrated to improve cognitive function in Alzheimer’s disease mouse models by accelerating molecular transportation in the brain ECS. This suggests that PBM may have a potential role in the accumulation of pathobiomolecules in the brain ECS following ischemic stroke. In this study, we developed a PBM therapy apparatus with custom parameters. By evaluating the treatment effect, we identified that 755 nm was the optimal light wavelength for ischemic stroke in rats with transient middle cerebral artery occlusion/reperfusion. Extracellular diffusion and interstitial fluid (ISF) drainage were measured using a tracer-based magnetic resonance imaging method. Our results showed that PBM accelerated molecular transportation in the brain ECS and ISF drainage, promoting the clearance of pro-inflammatory cytokines and reducing the deposition of pathological proteins. Consequently, the infarct volume decreased and neurological cognitive function was improved. Besides, the acceleration of ISF drainage was achieved by reducing expression and restoring polarization of aquaporin 4 (AQP4) in the peri-infarct area. In summary, we demonstrated that PBM could alleviate ischemia–reperfusion injury and prevent post-stroke cognitive impairment by accelerating molecular transportation in the brain ECS, paving a pathway for ischemic stroke treatment via the light–ECS interaction.

## Introduction

Stroke remains a leading cause of death and long-term disability worldwide [1]. Many stroke survivors suffer from motor, cognitive, and psychiatric impairments, with over one-third developing post-stroke cognitive impairment (PSCI), severely affecting their quality of life [2]. The complex pathophysiological mechanisms of stroke and PSCI involve overactivation of microglia and astrocytes and release of a large amount of pro-inflammatory cytokines that destruct the blood–brain barrier (BBB), damage neurons, and aggravate brain injury [3,4]. In addition, the accumulation of neuropathological substrates, such as parenchymal amyloid-β (Aβ) plaques and neurofibrillary tau tangles, is associated with cognitive decline post-stroke [5,6]. Recent studies have demonstrated that the accumulation of pro-inflammatory cytokines in the extracellular space (ECS) plays a critical role in neurological damage and the exacerbation of conditions following ischemic events [7,8] In addition, impaired clearance of neuropathological substrates contributes to cerebral amyloid angiopathy and dementia [9], and reducing their accumulation in the brain ECS has been an important therapeutic strategy [7]. Comprising approximately 15% to 20% of total brain volume, the ECS is a critical microenvironment for neuronal survival and function [10]. The brain interstitial fluid (ISF) drainage within ECS, a recently elucidated network that is responsible for cerebrospinal fluid (CSF) circulation and waste clearance [11], facilitates the clearance of pathobiomolecules, including pro-inflammatory cytokines and neuropathological proteins [12]. The ECS and ISF function similarly to a lymphatic system and are recognized as key anatomical structures involved in cerebral clearance [13]. Previous studies have demonstrated that actively regulating the ECS to accelerate ISF drainage can effectively reduce inflammatory factors, thereby protecting the brain after stroke [7]. Therefore, facilitating the clearance of pathobiomolecules by accelerating ISF drainage in the brain ECS is a potential approach to treat ischemic stroke.

Photobiomodulation (PBM) has garnered considerable attention for its efficacy in treating conditions such as traumatic brain injury (TBI), Alzheimer’s disease (AD), stroke, wound healing, and spinal cord injuries and in mitigating side effects of cancer treatments [14–16]. Although the exact mechanisms of PBM are not yet fully elucidated, it is believed to modulate the production of various reactive oxygen species (ROS) and activates a variety of transcription factors of genes related to antioxidation, anti-inflammation, anti-apoptosis, proliferation, and migration functions [17,18]. Therefore, PBM shows promise as a novel nondrug and low-invasive treatment strategy for ischemic stroke. Numerous preclinical studies have found that PBM improves brain damage in animal models of ischemic stroke [14,19,20]. These studies have used light sources with fixed wavelengths and incident power to evaluate the biological effects of PBM. For instance, 630-nm laser markedly prevents the inflammatory response after acute ischemic stroke and inhibits microglia (Iba-1^+^) activation, thereby alleviating the development of cognitive impairment [14]. In addition, 808-nm light irradiation inhibits neurotoxic astrocyte polarization and protects neurons in both in vitro and in vivo stroke models [19]. Studies using wavelengths of 750 and 950 nm have found that PBM alleviates ischemia–reperfusion injury and induces a sustained reduction in infarct volume after ischemic stroke [20]. On this basis, clinical studies of PBM for stroke treatment have been conducted. A series of NeuroThera Effectiveness and Safety Trials (NESTs) using 808-nm light has explored the therapeutic effects of PBM on acute ischemic stroke [21–23]. The positive outcomes in NEST-1 [21] and the signal toward efficacy in NEST-2 [22] provided evidence of the potential benefits of PBM treatment for acute ischemic stroke. The large NEST-3 was terminated early after a futility analysis of 566 participants showed no clinical difference in the primary 90-d modified Rankin scale score between the transcranial laser therapy group and sham treatment group [23]. Moreover, 630- and 870-nm light has shown potential in improving neurological function and enhancing intrinsic brain network connectivity in patients with chronic stroke [24]. Further research and refinement of trial designs are needed to fully understand the potential benefits and limitations of PBM for the treatment of stroke. Despite these promising results, the mechanisms underlying PBM therapy for stroke remain incompletely understood. Notably, these studies have not taken into account the role of the brain ECS, which may be a key factor contributing to the challenges in clinical translation of PBM for ischemic stroke treatment. Recent research has revealed that PBM can accelerate ISF drainage by reducing the tortuosity and enhancing diffusion in the brain ECS [25,26]. Irradiation with 630-nm red light reverses the Aβ-impeded ISF flow and improves memory decline of APP/PS1 mice [27]. This suggested that an essential pathway by which PBM exerts its neuroprotective effects is through modulation of the brain ECS.

In this study, we hypothesized that PBM could treat ischemic stroke by accelerating brain ISF drainage in the ECS. Specifically, PBM could reduce inflammatory cytokines and neuropathological proteins, ultimately providing neuroprotection and preventing PSCI. To demonstrate this, we investigated the neuroprotective effects and ECS dynamics of laser at wavelengths of 638, 755, and 808 nm using the transient middle cerebral artery occlusion (tMCAO) model of ischemic stroke. We optimized light stimulation parameters by comparing the effects among different near-infrared (NIR) light wavelengths. We demonstrated that PBM at 755 nm not only reduced the inflammatory response and ischemia–reperfusion injury but also cleared neuropathological proteins, which improved neurocognitive function during post-stroke sequelae period, preventing PSCI via accelerated ISF drainage by modulating ECS properties. This ISF regulation was achieved as the 755-nm laser alleviated astrocyte activation and altered the expression of aquaporin 4 (AQP4). These findings highlight the crucial role of ECS in PBM neuroprotection and hold promise for treating stroke.

## Materials and Methods

### Experimental design

**Fig. 1.**
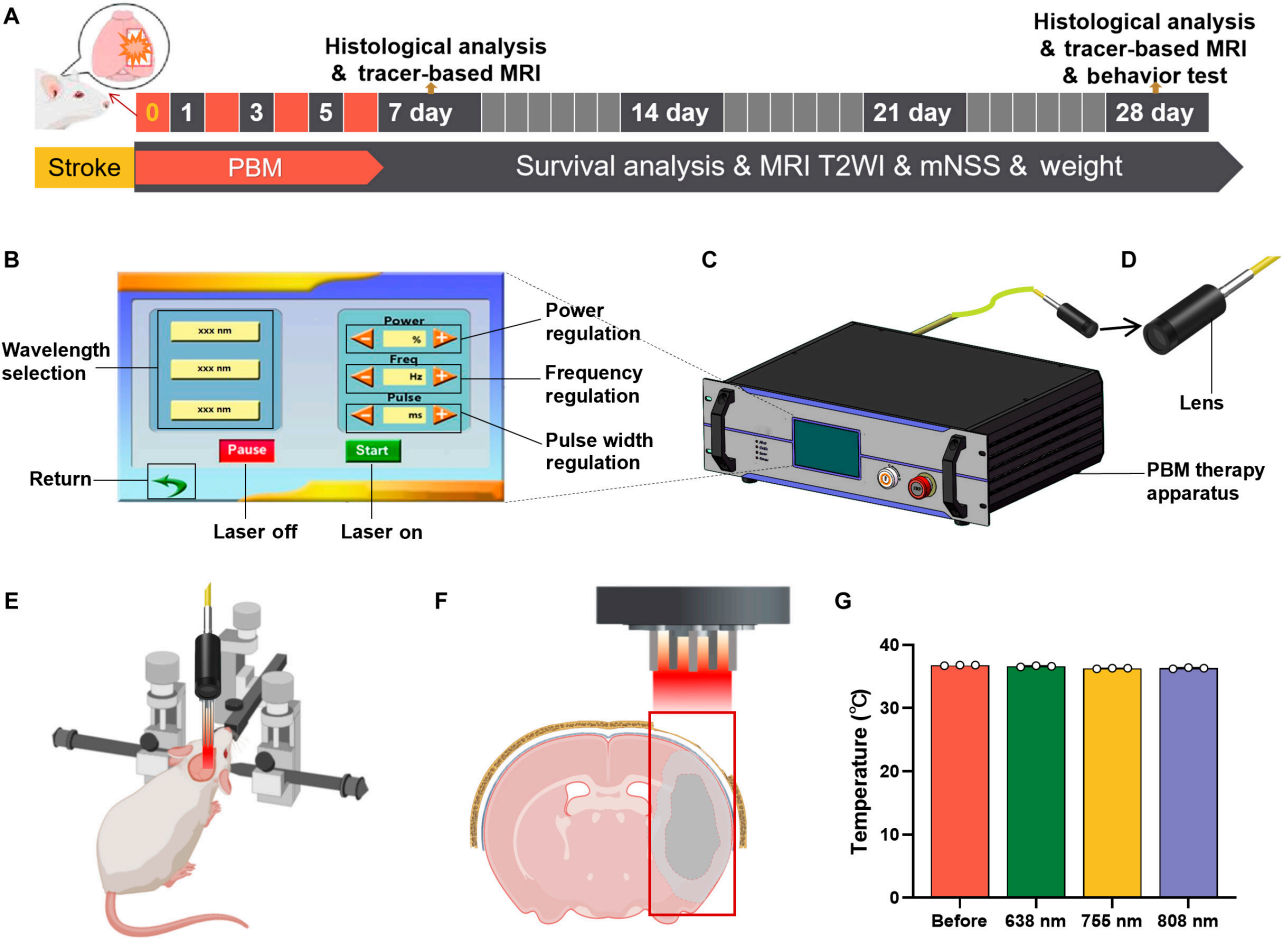
Experimental design and PBM therapy apparatus developed for tMCAO rat treatments. (A) Experimental schematic diagram. (B to D) PBM therapy apparatus (C) including a touchscreen control (B) and a lens (D). (E) The rats were fixed in a stereotaxic apparatus during PBM therapy. (F) Schematic illustration of the lens placed outside the skull, above the brain. The red box delineates the proposed treatment area. (G) Temperature: Surface temperature of the irradiated area of the rat brain before and after phototherapy. The surface temperatures of the irradiated area in the rat brain, measured at wavelengths of 638, 755, and 808 nm, showed no significant difference compared to pre-irradiation levels. Data are represented as mean ± SEM. *n* = 3 per group.

The experimental schematic diagram is shown in Fig. 1A. The tMCAO rats were randomly assigned to 5 groups: (a) the tMCAO group; (b) the tMCAO + sham illumination group (tMCAO + Sham group), and 3 tMCAO + PBM groups: (c) tMCAO + 638 nm group; (d) tMCAO + 755 nm group; and (e) tMCAO + 808 nm group. The 3 tMCAO + PBM groups received laser irradiation at their corresponding wavelengths, which had no effect on brain surface temperature before or after irradiation (Fig. 1G). Additionally, the Normal group was regarded as the control group for cognitive behavioral testing and tracer-based magnetic resonance imaging (MRI).

On days 1, 3, 7, 14, 21, and 28 post-tMCAO, the survival status of the rats was recorded, and body weights were measured and documented. The modified neurological deficit scores (mNSS) was conducted on days 1, 3, 7, 14, 21, and 28 post-tMCAO. Open-field test (OFT) and new object recognition (NOR) test were conducted on day 28 post-tMCAO. Morris water maze (MWM) test were conducted continuously for 6 d from day 23 to day 28 post-tMCAO. Rats were scanned with MRI T2-weighted imaging (T2WI) on days 1, 3, 7, 14, 21, and 28 after stroke. On days 7 and 28 post-tMCAO, rats were evaluated with tracer-based MRI scans. Rats were sacrificed on either day 7 or 28 after stroke. The brains were promptly dissected and then stored at −80 °C or fixed in 4% paraformaldehyde (PFA) for subsequent analysis.

### PBM therapy apparatus and procedures

We developed a PBM therapy apparatus to identify optimal parameters for PBM therapy in ischemic stroke (Fig. 1B to D), which is potentially adaptable for application in other diseases. This apparatus is designed with adjustable parameters, including wavelength (638 ± 5 nm, 755 ± 5 nm, 808 ± 3 nm), power (0 to 1 W), frequency (1 to 60 Hz), and pulse width (1 to 999 ms) (Honglanguangdian, Beijing, China).

The PBM therapy apparatus integrates a laser, driving circuit, control circuit, and cooling system into a single unit (Fig. 1C), with touchscreen control (Fig. 1B). Three semiconductor laser emitters, each with distinct wavelength, are housed in a metal enclosure, with fiber coupling used for light output. The wavelengths of 638 ± 5 nm, 755 ± 5 nm, and 808 ± 3 nm correspond to fiber core diameters of 105, 200, and 200 μm, respectively, with all fibers measuring 1 m in length. A uniform numerical aperture (NA) of 0.22 is maintained across all fibers. The fiber ends are equipped with SMA905 connectors, and the fibers are protected by metal inner armor tubes. The laser is coupled with an adjustable output lens, allowing for modulation of the spot size through lens rotation and movement (Fig. 1D). Additionally, a filter integrated into the lens dust cap enables adjustment of the laser power density. The entire system employs a forced air cooling mechanism for heat dissipation, requiring sufficient space around the laser for proper ventilation. The device is powered by an AC 220 V supply.

PBM therapy involves multiple complex parameters, including wavelength, light intensity, and exposure time [28]. In this study, we focused on optimizing wavelength due to its notable impact on treatment outcomes [14,20,29]. The tissue penetration properties of the 600- to 880-nm light, together with its high efficacy, have generally made it the most popular wavelength range [30]. Kim et al. [14] have compared the effects of PBM therapy for ischemic stroke using different wavelengths, including 630, 850, and 940 nm. Among these, 630 nm has shown superior outcomes in promoting functional recovery compared to the other 2 wavelengths. PBM therapy using 940 nm also results in some functional improvement post-stroke, although the effect is less pronounced than that observed with 630 nm. Notably, 808 nm is currently the most widely used wavelength in stroke research [15]. However, recent evidence suggests that 760 nm may offer even greater therapeutic benefits than 808 nm [20]. Given these considerations, our study focuses on investigating the effects of 630-, 808-, and 755-nm wavelengths. Regarding light intensity and exposure time, we selected conditions based on literature and preliminary experiments [30,31]. A beneficial power density range of approximately 10 to 25 mW/cm^2^ has been reported for PBM studies in thin-skull animal models [14]. There are currently no unified guidelines regarding the optimal duration and intervals for PBM treatments. Studies have shown that a single large dose of irradiation is less effective than multiple smaller doses administered [32]. For acute brain conditions such as ischemic stroke, repeated treatments over 3 to 7 d are typically required [14,15], whereas chronic conditions like neurodegenerative diseases may need longer treatment periods (1 to 2 months) [17,27]. Additionally, it is generally believed that pulsed light is superior to continuous light for PBM applications [31].

Based on the aforementioned reports, we have chosen the following PBM procedures: For the tMCAO + PBM group and the tMCAO + Sham group, after the insertion and fixation of the filament (for more information on the “tMCAO model”), the rats were repositioned prone and restrained (Fig. 1E). The midline incision was performed on the scalp after routine skin preparation. The superficial soft tissues were removed until the skull was adequately exposed. A circular cranial window of 0.4 cm diameter was selected in the right parietal bone, and the skull was ground with a bone drill until the pial vessels were clearly visible (Fig. 1F). For the tMCAO + PBM group rats, the laser emission probe was fixed 5 cm above the cranial window, and 10-min irradiations were administered upon removal of the suture and at fixed times on days 1 to 6 post-tMCAO. The diameter of the light spot on the surface of the skull is 4 mm. The power density of the laser irradiation on the cranial window surface was 20 mW/cm^2^, and the pulse frequency was 60 Hz. The rats in the tMCAO + Sham group experienced the same procedure as the treatment group, while the PBM therapy apparatus remained off.

### Animals

Sprague–Dawley rats (280 to 320 g, male) were used in this experiment. This study has been approved by the Peking University Biomedical Ethics Committee (approval number: LA2021507). The rats were housed under a 12-h light/dark cycle at a constant temperature of (22 ± 1) °C, with free access to food and water. All surgeries were performed under anesthesia.

### tMCAO model

This research utilized the tMCAO model, which is the most commonly employed animal models for studying ischemic stroke. Rats with mNSS indicating no apparent postoperative deficits (mNSS = 0 to 1) were excluded. Throughout the operation, isoflurane (RWD Life Science, Shenzhen, China) was used as gas anesthesia. Routine disinfection of the neck skin was performed, followed by a median cervical incision to expose and isolate the vessels. After that, the external carotid artery and the common carotid artery were ligated. A small incision was made in the external carotid artery. Then, a monofilament nylon suture with a 0.2-mm-diameter silicone rubber-coated tip (Jialing, Guangzhou, China) was used to insert into the internal carotid artery. After 90 min, the sutures were gently removed and the skin was sutured.

### Temperature measurement

The surface temperature of the cranial window was assessed with an infrared thermometer (KF-HW-002, Cofoe, Hunan, China) both before and after laser irradiation, with 3 repeated measurements at the same location.

### Cognitive behavioral testing

#### mNSS

The mNSS [33] includes tests for motor function, sensation, reflexes, and balance beam performance, with a maximum score of 18. An inability to perform a task or lack of a reflex adds 1 point, scores of 13 to 18 indicate severe injury, scores of 7 to 12 indicate moderate injury, and scores of 1 to 6 indicate mild injury. All mNSS assessments were conducted independently by 2 evaluators who were blinded to the experimental groups. Both evaluators underwent specialized training and reached consensus on scoring criteria through multiple pre-tests prior to the formal experiments. The final results were calculated as the average of the scores provided by the 2 evaluators.

#### OFT

Sensory-motor deficits were assessed through the OFT. Rats were placed in the open field (100 cm × 100 cm × 40 cm), and their movements were recorded for 10 min. Subsequently, the data were analyzed using Labmaze V3.0 software for animal behavioral tracking.

#### MWM

Long-term cognitive deficits are assessed through the MWM test. During the training phase, all rats underwent 4 trials per day. Each rat was given 60 s to locate the platform, and each trial started from a different quadrant. If the rat found the platform before the 60-s cutoff time, it was allowed to stay for 5 s. In case of failure, the rat was guided to rest on the platform for 20 s, and the latency was recorded as 60 s. There was a 30-min interval between trials, and this continued for 5 d. The latency to reach the platform and swimming paths of each rat were recorded, and the decrease in latency to reach the platform during the training process reflected learning and memory. A probe test, which allows rats to find the platform for 60 s, was conducted after removing the platform on the last day. The escape latency to find the platform, swimming path length, time spent in the target quadrant, and number of platform crossings were tracked and analyzed using ZS-Morris software.

#### NOR

The NOR test relies on rodents’ natural inclination to explore novelty. The rats were placed in an unoccupied area and given an opportunity to explore freely for a duration of 10 min throughout the habituation phase. After 1 d, one object was placed in corners, and another was placed in the centrosymmetry of the arena. The 2 objects were identical and nontoxic; each rat was allowed to freely explore for 10 min. Two hours later, one of the previously explored objects was replaced with a novel object. Rats were allowed to explore the arena for another 10 min to test their preference for the new object. The exploration process of the rats was recorded throughout and analyzed using Labmaze V3.0 software for animal behavioral tracking. The preferred object was calculated as follows: *DI* (discrimination index) = (*T*_novel_ − *T*_familiar_)/(*T*_novel_ + *T*_familiar_), where *T*_novel_ and *T*_familiar_ represented the exploration time while testing the novel and familiar objects, respectively.

### MR T2WI scanning and image processing

Rats were scanned in the prone position using a 3.0T Siemens trio MRI (MagnetomTrio, Siemens Medical Solutions, Erlangen, Germany). The main scanning parameters were as follows: repetition time = 4,200 ms, echo time = 83 ms, field of view = 4 cm × 3 cm, matrix = 384 × 320, slice thickness = 1 mm, number of slices = 24. Three-dimensional (3D) slicer was used to calculate the volume of brain edema and cerebral infarction. We corrected the swelling according to the method proposed by Gerriets et al. [34]. The specific formula used was as follows: Brain lesion volume (BLV) = [(contralateral volume − (ipsilateral volume − infarct volume))]/2 × contralateral volume × 100%. Brain swelling volume (BSV) = Ipsilateral brain volume/Contralateral brain volume × 100%.

### Tracer-based MRI

#### Stereotaxic intraparenchymal injection

The gadolinium diethyltriaminepentaacetic acid (Gd-DTPA)-enhanced MRI scanning technique [35], independently developed by our laboratory, was utilized to measure the distribution of contrast agents in the brain ECS. Gd-DTPA was injected into the right caudate nucleus of rats via stereotaxic intraparenchymal injection, following the procedure outlined previously [7]. In accordance with the coordinates outlined in *The rat brain in stereotaxic coordinates, sixth edition* (Paxinos and Waston, 2007, Academic Press), a stereotaxic apparatus was used to identify the right caudate nucleus. The specific stereotaxic coordinates were as follows: 1 mm anterior to the bregma, 3 mm lateral to the midline, and 6 mm in depth. A total volume of 2 μl of 10 mM tracer was administered at a rate of 0.2 μl/min. To facilitate this, a cranial burr hole was created at the designated location, allowing the microinjection needle to penetrate the brain parenchyma. The tracer was subsequently injected directly at the target site. MRI images were obtained using a MagnetomTrio scanner (Siemens Medical Solutions, Erlangen, Germany), paired with a wrist coil. T1-weighted magnetization was used to acquire rapid gradient-echo sequences. The scanning protocols and subsequent image processing were carried out as described in earlier studies [7].

#### Cisterna magna injection tracer

Images were acquired using a 3.0T MRI scanner (MR750, GE, USA).

Pre-scan: The experiment was performed using a 3.0T GE750 MR, anesthesia was administered by intraperitoneal injection, and the deeply anesthetized rat was placed in the accompanying coil (coil type) for a pre-scan. The scanning parameters are as follows: repetition time: 9,800 ms, echo time: 4.4 ms, field of view: 6.4 cm × 6.4 cm, matrix: 160 × 160, slice thickness: 0.4 mm, bandwidth: 195.4 Hz/px, flip angle: 9°, phase encoding steps: 160, number of slices: 52, number of averages: 2.

Subarachnoid space injection: After the pre-scan, the rat was removed from the MRI room and placed prone on the surgical table, and the hair at the occipital region was shaved and locally disinfected. A horizontal incision was made behind the rat’s ears using surgical scissors, followed by a vertical incision along the midline, creating a T-shaped incision. Then, using forceps, the trapezius muscle was torn from the incision site, blunt dissection of the deeper levator muscle was performed, and the medial half of its tendon was cut. Hemostatic forceps were used to hold the cut end of the levator muscle, fully exposing the field of operation. Starting approximately 3 mm below the occipital protuberance on the midline, the injection needle was moved downward along the occipital bone until the tip touched the atlantooccipital membrane. The direction of the needle was adjusted so that the slope faced upward, applying appropriate force. Once a sense of loss was felt, the needle advancement was immediately stopped, indicating that the needle had penetrated the dura mater and entered the cerebellomedullary pool. The needle was then secured in place. Then, administration began at a constant infusion rate of 2 μl/min, delivering 21 mM Gd-DTPA into the cerebellomedullary pool within 20 min.

Dynamic MRI scanning: To dynamically monitor the flow of the tracer, the T1WI scanning time series was conducted at 0, 15, 30, 45, 60, 75, 90, 120, 150, 180, and 240 min post-injection. Throughout the scanning process, attention was paid to constantly monitor the rat’s vital signs. During this period, the rat was placed on an electric blanket to maintain stable body temperature as much as possible. At the same time, anesthesia was supplemented according to individual differences to ensure that the rat remained in a state of deep anesthesia throughout the entire experiment.

Post-processing of MR images: First, the T1WI images were converted to the 3D NIfTI image format using the python module dicom2nifti. Second, affine registration transformation was used to align the images at each time point to the baseline image taken before drug administration to correct for image misalignment caused by head movement. Third, the intensity nonuniformity of the baseline image was estimated using the python module SimpleITK, and baseline intensity nonuniformity correction was performed on all volumes to ensure consistency. Further smoothing of the corrected images was done using a Gaussian kernel function with a full width at half maximum isotropic Gaussian voxel image intensity of 0.1 mm to reduce noise and smooth the image details. To obtain the signal enhancement regions, the difference between each time point image and the baseline image was calculated using the following expression:

$$C\left(x,\;y,\;z\right)=I\left(x,\;y,\;z\right)-B\left(x,\;y,\;z\right)\;\left(1\right)$$

where *C* represents the subtraction image after the difference, *I* is the series of time images after drug administration, *B* is the baseline image before drug administration, and (*x*, *y*, *z*) is the voxel position. On the baseline T1WI image, registration with a standard mouse brain atlas was performed, regions of interest were selected, and mask images were acquired to extract the regions of interest on each time point image, calculate the average signal intensity, and convert it to the percentage signal enhancement relative to the baseline image.

### Cytokine quantification by ELISA

The supernatant was harvested after homogenizing the ischemic penumbra tissue with phosphate-buffered saline (PBS). The pro-inflammatory cytokines IL-1β, IL-6, and tumor necrosis factor-α (TNF-α) were detected in the samples using enzyme-linked immunosorbent assay (ELISA) kits (Rat IL-1 beta ELISA Kit, GER0002-48T; Rat IL-6 ELISA Kit, GER0001-48T; Rat TNF-alpha ELISA Kit, GER0004-48T, Servicebio, Wuhan, China). Absorbance was measured at 450 nm using a microplate reader.

### Immunofluorescence staining and histological analysis

The fixed brains were embedded in paraffin and cut into 6 μm. The brain sections were incubated overnight at 4 °C with primary antibodies (anti-NeuN, anti-GFAP, anti-Iba-1, anti-tau, anti-p-tau, anti-Aβ, anti-AQP4, anti-GLUT1) (Servicebio, Wuhan, China). After washing with PBS, the brain sections were incubated with appropriate secondary antibodies. The process lasted for an hour at room temperature. Images were acquired by upright fluorescence microscope (NIKON ECLIPSE C1, Japan) with a 40× objective at identical settings for all conditions. ImageJ (National Institutes of Health, USA) was used to analyze the images.

#### Analysis of AQP4 localization

The localization of AQP4 was analyzed using costained images of AQP4, GLUT1, and GFAP. The specific analysis process was performed according to Harrison et al. [36] The median immunofluorescence intensity of AQP4 in the perivascular region was used as the threshold for measuring AQP4 polarization. Threshold analysis was then applied to measure the percentage area of AQP4 fluorescence intensity greater than or equal to the threshold (AQP4 percentage area). Polarization is expressed as the percentage area of the region below the perivascular area with AQP4 fluorescence intensity (100-AQP4 percentage area).

AQP4 localization at the perivascular terminal feet and glial limitans was evaluated by measuring the pixel intensity of GLUT1 and AQP4 immunoreactivity in blood vessel cross-sections from the brain regions of interest. Six vessels were randomly selected in the peri-infarct area of each rat for analysis. The fluorescence intensity of GLUT1 and AQP4 was measured along a 200-pixel line perpendicular to the vessel, and the intensity values were used to create a linear profile extending from the brain tissue, across the vessel, and back to the surrounding brain tissue.

### Statistical analysis

Statistical analyses were performed using GraphPad Prism 9 (version 9.5.0, GraphPad Software Inc., La Jolla, CA). Data are presented as mean ± standard error of the mean (SEM). Parametric or nonparametric tests were chosen based on normality test results. A one-way analysis of variance (ANOVA) was used to compare multiple groups, followed by Tukey’s honest significant difference (HSD) test for pairwise comparisons. For data with nonnormal distribution, Kruskal–Wallis tests were applied. Log-rank tests were used for survival analysis. Statistical significance was defined as *P* < 0.05.

## Results

### Comparison of the effects of different PBMs on alleviating ischemia–reperfusion brain injury in tMCAO rats

**Fig. 2.**
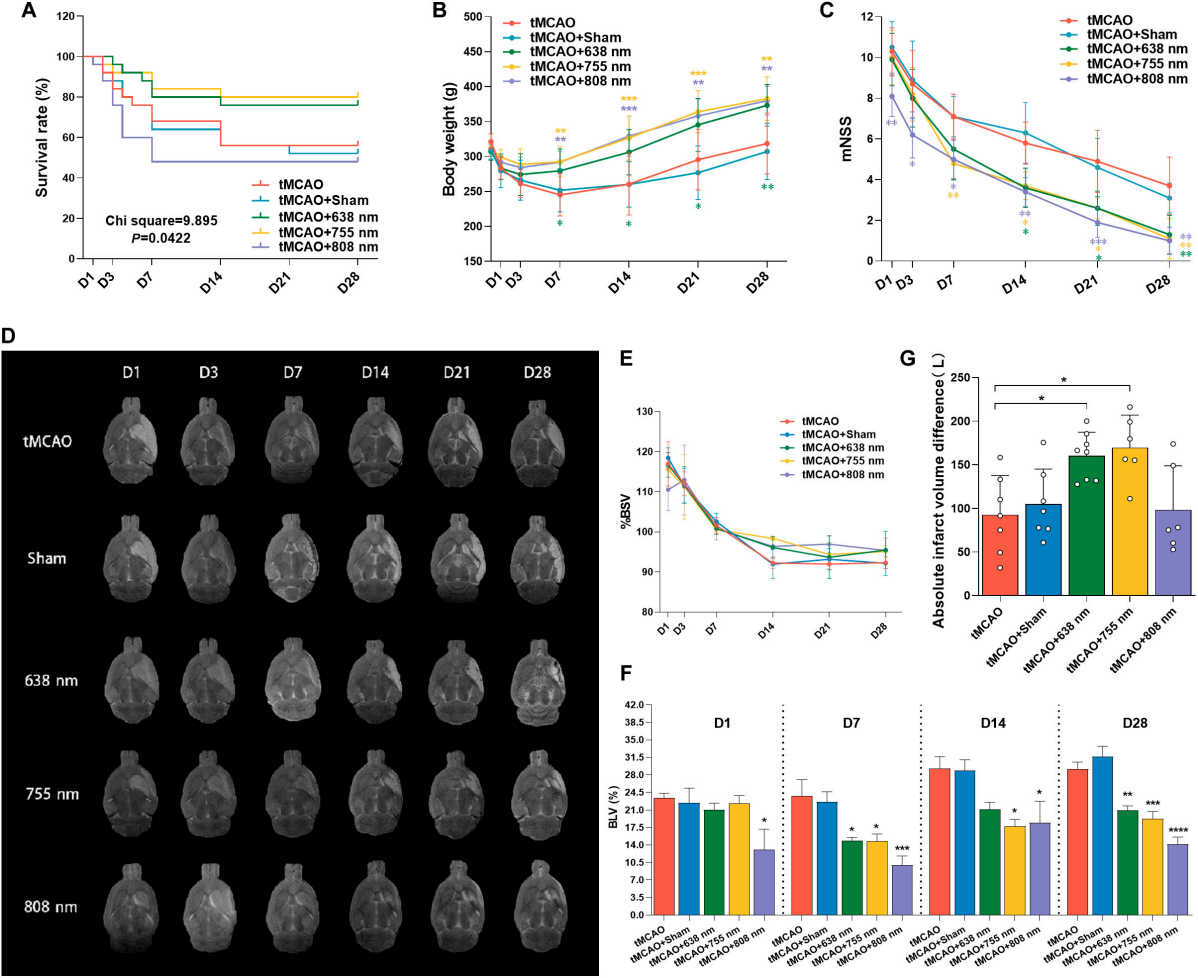
PBM alleviated neurological deficits and ischemic injury in tMCAO rats. (A) Survival rate: Recording of survival status of all model rats, subjecting them to survival analysis. (B) Body weight: Body weight of rats surviving up to day 28 in each group. (C) mNSS: mNSS of rats surviving up to day 28 in each group. (D) Representative MR T2WI images in different groups on days 1, 3, 7, 14, 21, and 28 post-stroke: The images for each group are selected from different time points of the same rat to illustrate the dynamic changes in an individual animal throughout the experimental period. (E) BSV of each group on days 1, 3, 7, 14, 21, and 28 post-stroke. (F) Brain ischemic lesion volume of each group on days 1, 7, 14, and 28 post-stroke. (G) Absolute value of the difference in ischemic lesion volume between day 1 and day 28 post-stroke in each group. In (D) to (F), *n* = 6 to 8 per group. Data are presented as mean ± SEM. *P < 0.05, **P < 0.01, ***P < 0.001, or ****P < 0.0001.

We calculated the survival rates of the tMCAO rats in each group after stroke. Furthermore, we analyzed the body weights at days 1, 3, 7, 14, 21, and 28 post-stroke of the tMCAO rats that survived up to 28 d in each group as well as their mNSS at these time points. Comparing the tMCAO + Sham group with the tMCAO group after stroke, no significant differences were observed in survival rates, body weight, or mNSS (Fig. 2A to C). However, compared to the tMCAO group and the tMCAO + Sham group, the tMCAO + 755 nm group and the tMCAO + 638 nm group showed a slight increase in survival rate (Fig. 2A). In contrast, the tMCAO + 808 nm group experienced a decrease in survival rate, with a substantial number of rats dying during the PBM therapy period (chi square = 9.895, *P* = 0.0422) (Fig. 2A). From day 7 onward, the tMCAO + 755 nm group showed improved body weight and mNSS compared to the tMCAO group and the tMCAO + Sham group (Fig. 2B and C). Compared to the tMCAO group and the tMCAO + Sham group, the tMCAO + 638 nm group exhibited higher body weights and lower mNSS scores, although these differences were not as pronounced as those observed in the tMCAO + 755 nm group (Fig. 2B and C). In the tMCAO + 808 nm group, only the rats that survived up to 28 d had their body weights and mNSS scores fully recorded. These rats initially showed milder cerebral infarction, as indicated by their lower mNSS scores on day 1. The body weights and mNSS scores of the tMCAO + 808 nm group included in the statistical analysis were significantly better than those of the control groups (Fig. 2B and C).

To investigate the impact of different wavelength light exposure on ischemia–reperfusion injury in tMCAO rats, MRI T2WI was used to observe dynamic changes in the brain (Fig. 2D). It was observed that tMCAO rats developed cerebral infarction lesions in the middle cerebral artery region, accompanied by acute cerebral edema manifestations such as the midline structure shifts, reduced ventricular compression, and tissue swelling. Chronic phases exhibited brain atrophy. The brain swelling/shrinking volume (BSV) was calculated at various time points post-stroke. As shown in Fig. 2E, on days 1 and 3, brain edema was evident in all groups (BSV > 100%), with no notable differences in volume among them. Subsequently, edema began to subside. From day 14, brain atrophy became apparent (BSV < 100%). By day 28, the tMCAO + 755 nm group exhibited less brain atrophy compared with the control group, although the differences were not statistically significant. Meanwhile, changes in the BLV were quantified (Fig. 2F). No significant differences in BLV were shown between the tMCAO group and the tMCAO + Sham group. Throughout the observation period, BLV in the tMCAO + 808 nm group consistently remained smaller than that in the tMCAO group (*P* < 0.05, *P* < 0.001, *P* < 0.05, *P* < 0.0001 versus tMCAO on days 1, 7, 14, 28). On day 1, BLV did not significantly differ among the remaining groups. By day 7, BLV in the tMCAO + 755 nm group was lower than that in the tMCAO group and the tMCAO + Sham group, and by day 28, BLV in the tMCAO + 755 nm group decreased by 45.3% (*P* > 0.05, *P* < 0.05, *P* < 0.05, *P* <0.001 versus tMCAO on days 1, 7, 14, 28). To better describe the treatment effect, the differences in absolute infarct volume between each group on days 1 and 28 were also compared (Fig. 2G). The results showed that the absolute infarct volume was reduced in rats subjected to tMCAO + 638 nm and tMCAO + 755 nm compared with the control group (*P* < 0.05, *P* < 0.05 versus tMCAO). Compared to the tMCAO and tMCAO + Sham groups, rats in the 808-nm laser irradiation group exhibited no significant differences (*P* > 0.05, *P* > 0.05 versus tMCAO, tMCAO + Sham).

To further clarify the protective effects of different wavelength light exposure on tMCAO rats, hematoxylin and eosin (H&E), Nissl, terminal deoxynucleotidyl transferase-mediated deoxyuridine triphosphate nick end labeling (TUNEL), and NeuN staining were used to evaluate neuronal damage and survival on day 7 after tMCAO (Figs. S1 and S2). The results suggest that 755-nm laser substantially reduces neuronal damage.

Collectively, these results suggest that PBM therapy, particularly using a 755-nm wavelength laser, applied to the ipsilateral brain in ischemic cases, not only reduces brain injury but also facilitates neurological functional recovery following ischemic stroke.

### The 755-nm laser alleviates the inflammatory response following ischemia–reperfusion injury

**Fig. 3.**
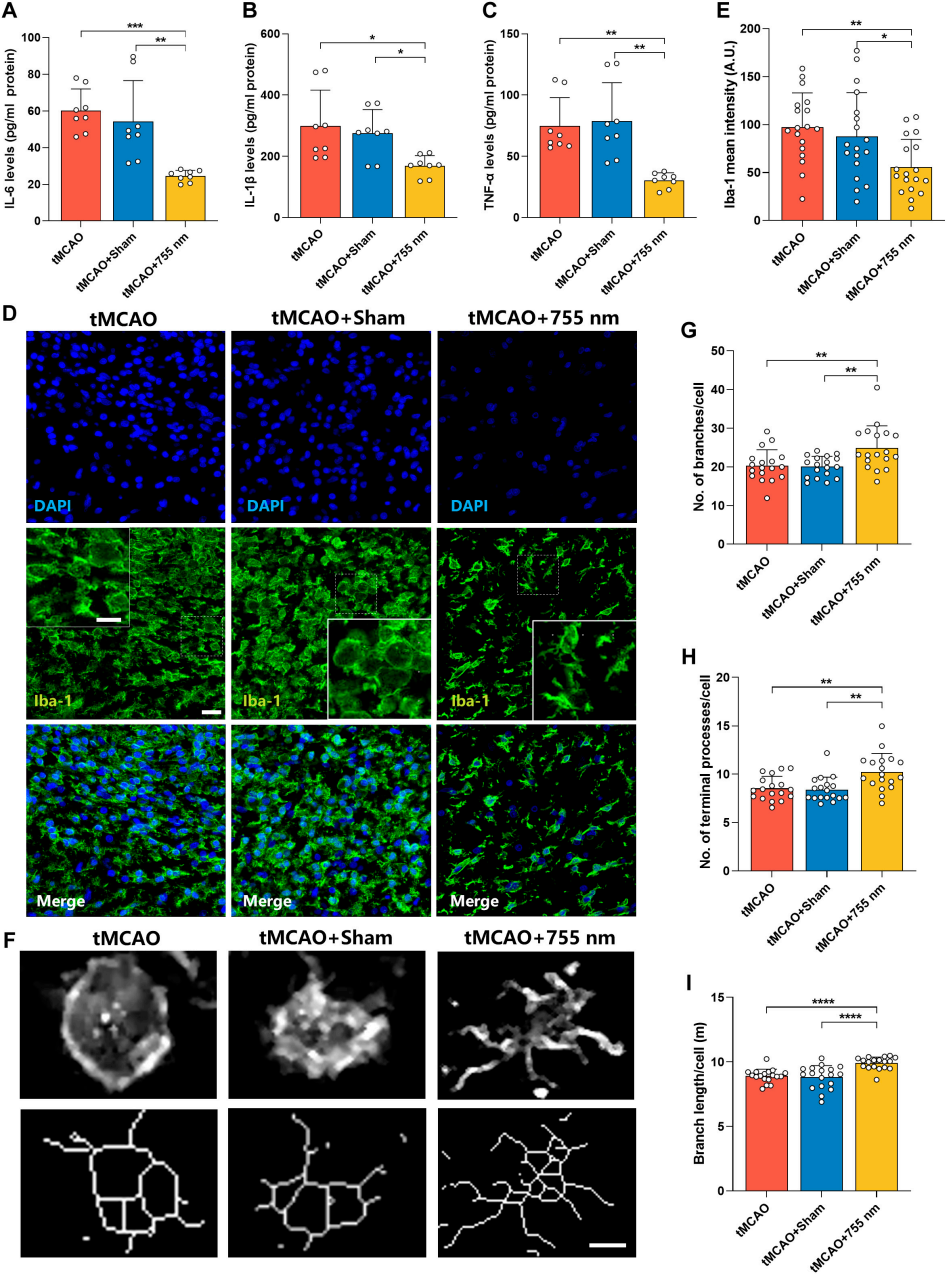
Laser irradiation (755 nm) alleviated the inflammatory response following ischemia–reperfusion injury after ischemic stroke. (A to C) Levels of pro-inflammatory cytokines in the infarcted side among different groups monitored using ELISA. Levels of IL-6 (A), IL-1β (B), and TNF-α (C) in the infarcted side 7 d post-reperfusion. *n* = 3 per group. All experiments were independently repeated twice. All data are expressed as mean ± SEM. *P < 0.05, **P < 0.01, or ***P < 0.001. (D) Fluorescent images of microglia (Iba-1^+^) in the peri-infarct area 7 d after tMCAO. Iba-1^+^ (green) and 4′,6-diamidino-2-phenylindole (DAPI) (blue). Scale bar, 20 μm. Enlarged images show typical cellular morphology (scale bar, 10 μm). (E) Average fluorescence intensity of microglia (Iba-1^+^) in the peri-infarct area. (F) Topological skeletonized images of representative microglia from 3 groups. Scale bar, 5 μm. (G to I) Number of branches per cell (G), number of terminal protrusions per cell (H), and length of branches per cell (I) in 3 groups of rats 7 d post-tMCAO. In (D) and (E), *n* = 3 to 4 per group, with 6 random fields of view analyzed per rat in the peri-infarct area. In (F) to (I), *n* = 3 to 4 per group, with 6 randomly selected whole microglial cells analyzed per rat in the peri-infarct area. Data are expressed as mean ± SEM. *P < 0.05, **P < 0.01, ***P < 0.001, or ****P < 0.0001.

After ischemic injury, neuronal death triggers inflammation response characterized by focal activation of glial cells and infiltration of peripheral immune cells. Activated microglia secrete pro-inflammatory cytokines including TNF-α, interleukin-6 (IL-6), and IL-1β within the brain ECS. The expression levels of these pro-inflammatory cytokines among different groups were measured using the ELISA assay (Fig. 3A to C). Compared to the tMCAO and tMCAO + Sham groups, the levels of IL-1β (*P* < 0.001, *P* < 0.01 versus tMCAO, tMCAO + Sham), IL-6 (*P* < 0.05, *P* < 0.05 versus tMCAO, tMCAO + Sham), and TNF-α (*P* < 0.01, *P* < 0.01 versus tMCAO, tMCAO + Sham) in the tMCAO + 755 nm group were significantly reduced, indicating that 755-nm laser effectively decreases the accumulation of pro-inflammatory cytokines after ischemia–reperfusion, exhibiting notable anti-inflammatory effects.

The expression of the microglial marker Iba-1 and the activation of microglia were analyzed using immunofluorescence (Fig. 3D to I). The results showed that the expression of Iba-1 in the tMCAO + 755 nm group was significantly reduced compared to the tMCAO group after cerebral ischemia (*P* < 0.01, *P* < 0.05 versus tMCAO, tMCAO + Sham) (Fig. 3D and E). Activated microglia were identified by a deramified phenotype, characterized by retracted processes, enlarged cell bodies, and an increased capacity for phagocytosis. Further, morphological changes of microglia (Fig. 3F) revealed that on day 7 post-modeling, the inflammatory response was subsiding and light-modified morphological features were observed in microglia. Compared to the tMCAO and tMCAO + Sham groups, the number (*P* < 0.01, *P* < 0.01 versus tMCAO, tMCAO + Sham) and length (*P* < 0.01, *P* < 0.01 versus tMCAO, tMCAO + Sham) of microglia branches were significantly increased after treatment with 755-nm laser irradiation (Fig. 3G and I). The terminal processes per cell (*P* < 0.0001, *P* < 0.0001 versus tMCAO, tMCAO + Sham) were significantly reduced (Fig. 3H). These results suggest that 755-nm laser clearly alleviates the inflammatory response following ischemia–reperfusion injury after ischemic stroke.

### The 755-nm laser improves the level of neurological cognitive function

**Fig. 4.**
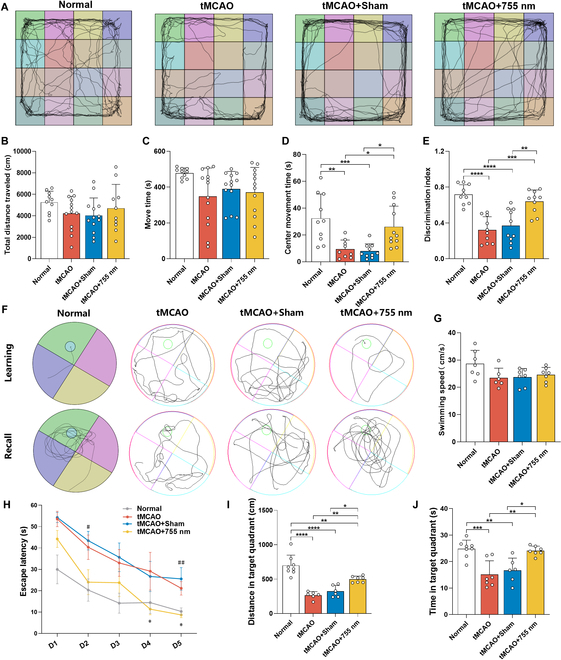
Laser irradiation (755 nm) improved neurocognitive function. (A) Typical move path in the open field test. (B) Total distance traveled. (C) Move time. (D) Time spent in the central region. (E) Discrimination index for new objects in the novel object recognition test. In (A) to (E), *n* = 10 to 14 per group. Data are expressed as mean ± SEM. *P < 0.05, **P < 0.01, ***P < 0.001, or ****P < 0.0001. (F) MWM swim path. (G) Swimming speed of rats during MWM. (H) Escape latency: The escape latency of tMCAO rats treated with 755-nm laser significantly shortened in the spatial navigation component. Statistically significant differences between tMCAO + Sham and tMCAO + 755 nm groups are indicated by the pound sign: ^#^*P* < 0.05 or ^##^*P* < 0.01. Statistically significant differences between tMCAO and tMCAO + 755 nm groups are indicated by asterisks: *P < 0.05. (I and J) Distance (I) and time (J) spent in the target quadrant during the probe trial without the platform for rats in each group. In (I) to (J), *n* = 6 to 8 per group. Data are expressed as mean ± SEM. *P < 0.05, **P < 0.01, ***P < 0.001, or ****P < 0.0001.

The mNSS results indicate improved neurological cognitive function in tMCAO rats following 755-nm laser treatment. Notably, differences were evident during the sequelae phase. To further elucidate the effect of the laser on cognitive impairment, we conducted the OFT, NOR, and MWM tests on day 28 post-stroke. In the OFT, a longer duration spent in the central area indicates lower anxiety levels and enhanced spatial cognition. Compared to the Normal group, rats in the tMCAO and tMCAO + Sham groups spent less time in the central area (*P* < 0.01, *P* < 0.001 versus tMCAO, tMCAO + Sham). The 755-nm laser significantly increased the time spent in the center (*P* < 0.05, *P* < 0.05 versus tMCAO, tMCAO + Sham) (Fig. 4A and D). In the NOR test, the discrimination index for novel object recognition ability assessment was used to assess short-term memory impairment. The Normal group demonstrated the highest ability to recognize new objects (*P* < 0.0001, *P* < 0.0001 versus tMCAO, tMCAO + Sham). Compared to the tMCAO and tMCAO + Sham groups, rats in the 755-nm laser irradiation group exhibited a significantly increased discrimination index (*P* < 0.001, *P* < 0.01 versus tMCAO, tMCAO + Sham) (Fig. 4E), indicating that the light treatment alleviated short-term memory deficits following ischemic stroke. No significant differences in average speed or activity time were observed among groups during the OFT and NOR tests, indicating that the results were independent of locomotor activity (Fig. 4A to C).

The superior performance of the 755-nm laser prompted further assessment of spatial learning and memory abilities in rats using the MWM test. Throughout the training process, all groups exhibited a trend of gradually decreasing escape latencies (Fig. 4F). The Normal group showed lower escape latencies compared to the other groups with ischemic stroke. As training progressed, the reduction in escape latencies became less pronounced in both the tMCAO and tMCAO + Sham groups, with no significant difference between them. Compared to the tMCAO group, rats in the tMCAO + 755 nm group displayed shorter escape latencies on days 4 and 5 (*P* < 0.05, *P* < 0.05 versus tMCAO). In addition, compared to the tMCAO + Sham group, the tMCAO + 755 nm group demonstrated reduced escape latencies on days 2 and 5 (*P* < 0.05, *P* < 0.01 versus tMCAO + Sham) (Fig. 4G). There were no significant differences in average swimming speed among the 4 groups (Fig. 4H). During the platform-free testing period, the time spent and swimming distance were measured in the quadrant where the platform was originally located, known as the target quadrant. Rats in the tMCAO + 755 nm group significantly increased both the time spent (*P* < 0.01, *P* < 0.05 versus tMCAO, tMCAO + Sham) and distance (*P* < 0.01, *P* < 0.05 versus tMCAO, tMCAO + Sham) traveled in the target quadrant (Fig. 4I and J).

These findings indicate that 755-nm laser irradiation significantly improved memory and spatial learning abilities in rats on day 28 post-stroke, alleviated anxiety, and prevented the occurrence of PSCI.

### The 755-nm laser alleviates the deposition of endogenous tau, p-tau, and Aβ in the brain of tMCAO rats

**Fig. 5.**
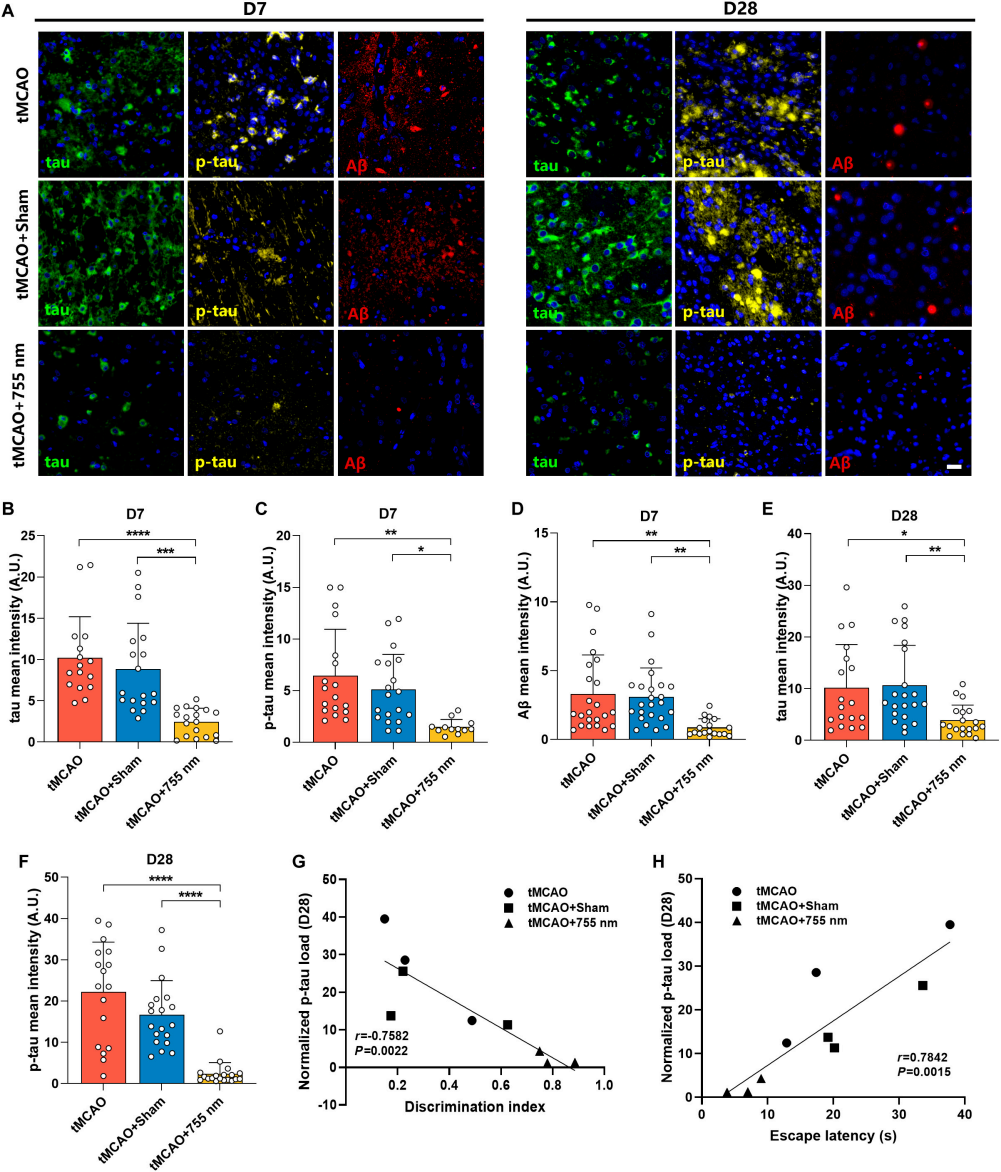
Laser irradiation (755 nm) reduced the deposition of endogenous tau, p-tau, and Aβ. (A) Immunofluorescence images of tau, p-tau, and Aβ in the peri-infarct area 7 or 28 d after tMCAO surgery. Tau (green), p-tau (yellow), Aβ (red), and DAPI (blue). Scale bar, 20 μm. (B to D) Mean fluorescence intensity of tau, p-tau, and Aβ in the peri-infarct area 7 d after tMCAO. (E and F) Mean fluorescence intensity of tau and p-tau in the peri-infarct area 28 d after tMCAO. In (B) to (F), *n* = 3 per group, with 6 random fields of view analyzed per rat in the peri-infarct area. Data are expressed as mean ± SEM. *P < 0.05, **P < 0.01, ***P < 0.001, or ****P < 0.0001. (G and H) Pearson correlation coefficient analysis of p-tau load in the peri-infarct area 28 d after tMCAO with the discrimination index in the NOR test (G) and escape latency in the MWM test (H).

The infarct location in the tMCAO model was relatively consistent. Besides the impact of infarct volume on post-stroke cognitive function, the role of neuropathological proteins remains to be elucidated. Therefore, we used immunofluorescence to further examine the deposition of tau, p-tau, and Aβ in the brains post-stroke. The area surrounding the infarction, most severely affected by the stroke, showed extracellular accumulation of tau and p-tau in the immunofluorescence staining images. A high Aβ load was observed, with prominent amyloid plaque formation (Fig. 5A). Statistical results revealed that on day 7 post-stroke, the load of tau (*P* < 0.0001, *P* < 0.001 versus tMCAO, tMCAO + Sham), p-tau (*P* < 0.01, *P* < 0.05 versus tMCAO, tMCAO + Sham), and Aβ (*P* < 0.01, *P* < 0.01 versus tMCAO, tMCAO + Sham) in the peri-infarct region of the tMCAO + 755 nm group was lower than in both the tMCAO and tMCAO + Sham groups (Fig. 5B to D). On day 28 post-stroke, the tau (*P* < 0.05, *P* < 0.01 versus tMCAO, tMCAO + Sham) and p-tau (*P* < 0.0001, *P* < 0.0001 versus tMCAO, tMCAO + Sham) load in the tMCAO + 755 nm group was significantly lower than in the other groups (Fig. 5E and F). The Aβ load on day 28 was low across all 3 groups with no significant differences among them (Fig. S2).

To determine the relationship between behavioral test performance and neuropathological substrate levels, we conducted Pearson correlation coefficient analyses. The p-tau load in the peri-infarct area on day 28 showed a negative correlation with the recognition index in the NOR test (Fig. 5G) and a positive correlation with the escape latency in the MWM (Fig. 5H). These results indicate that 755-nm laser irradiation can reduce the levels of tau, p-tau, and Aβ in the peri-infarct area of tMCAO rats, thereby improving their memory and cognitive abilities. Early administration of 755-nm phototherapy after stroke effectively prevents PSCI.

### The 755-nm laser modulates the ECS structure and ISF drainage in tMCAO rats

**Fig. 6.**
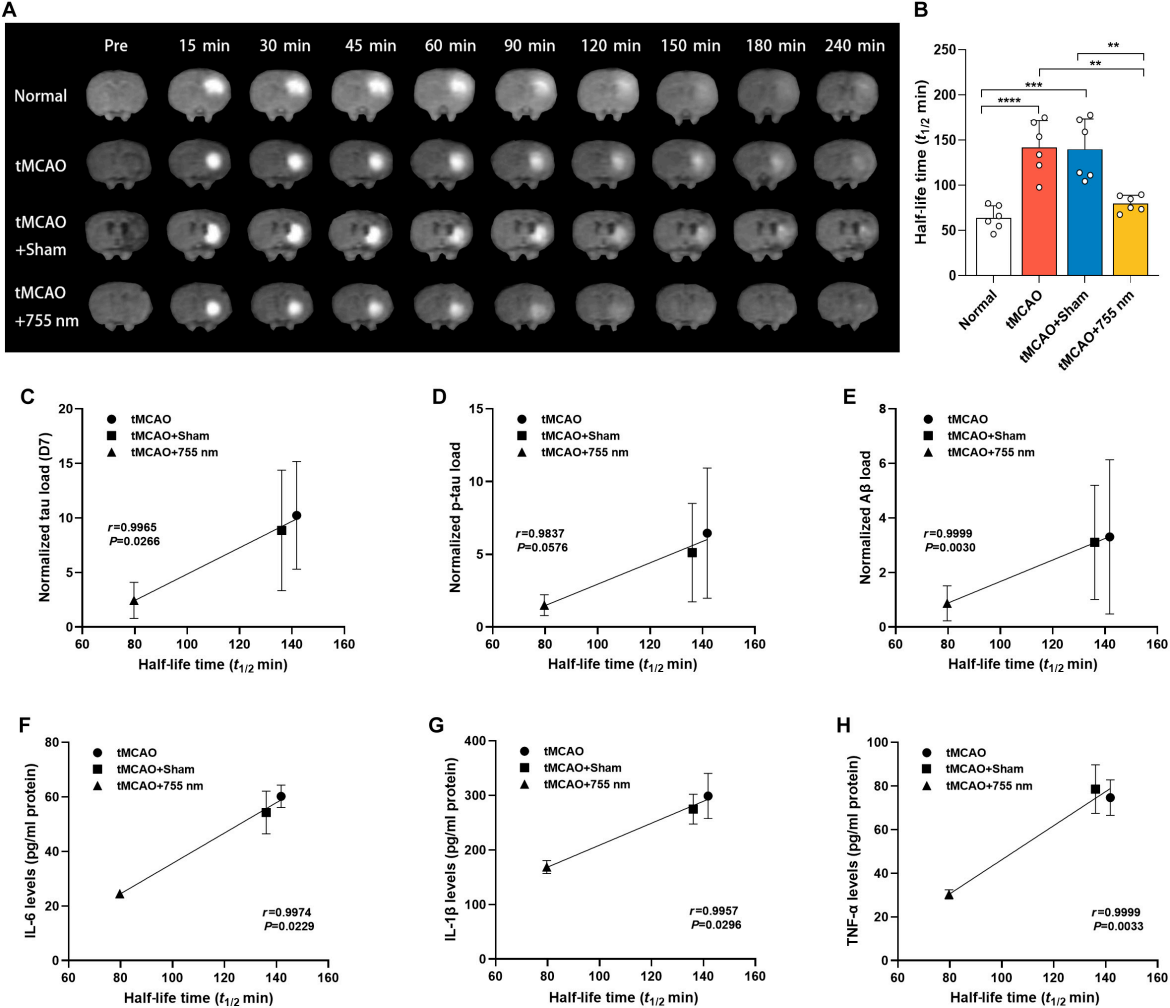
Laser irradiation (755 nm) accelerated the outflow of ISF in tMCAO rats. (A) Coronal MRI images of rats in each group 7 d after tMCAO and normal controls at 15, 30, 45, 60, 90, 120, 150, 180, and 240 min after tracer injection into the caudate nucleus area. After the tracer is injected into the caudate nucleus of the rats, a circular signal enhancement area becomes visible, which gradually expands over time along with a gradual reduction in signal strength, with differences in clearance rates among the different groups. (B) Statistical results for the ISF drainage parameter, half-life (*T*_1/2_), in the caudate nucleus area: Data are expressed as mean ± SEM. *P < 0.05, **P < 0.01, ***P < 0.001, *n* = 6. (C to E) Pearson correlation coefficient analysis of tau (C), p-tau (D), and Aβ (E) protein load in the peri-infarct region of rats 7 d after tMCAO surgery with *T*_1/2_. (F to H) Pearson correlation coefficient analysis of IL-6 (F), IL-1β (G), and TNF-α (H) levels in the infarct region of rats 7 d after tMCAO surgery with *T*_1/2_.

**Fig. 7.**
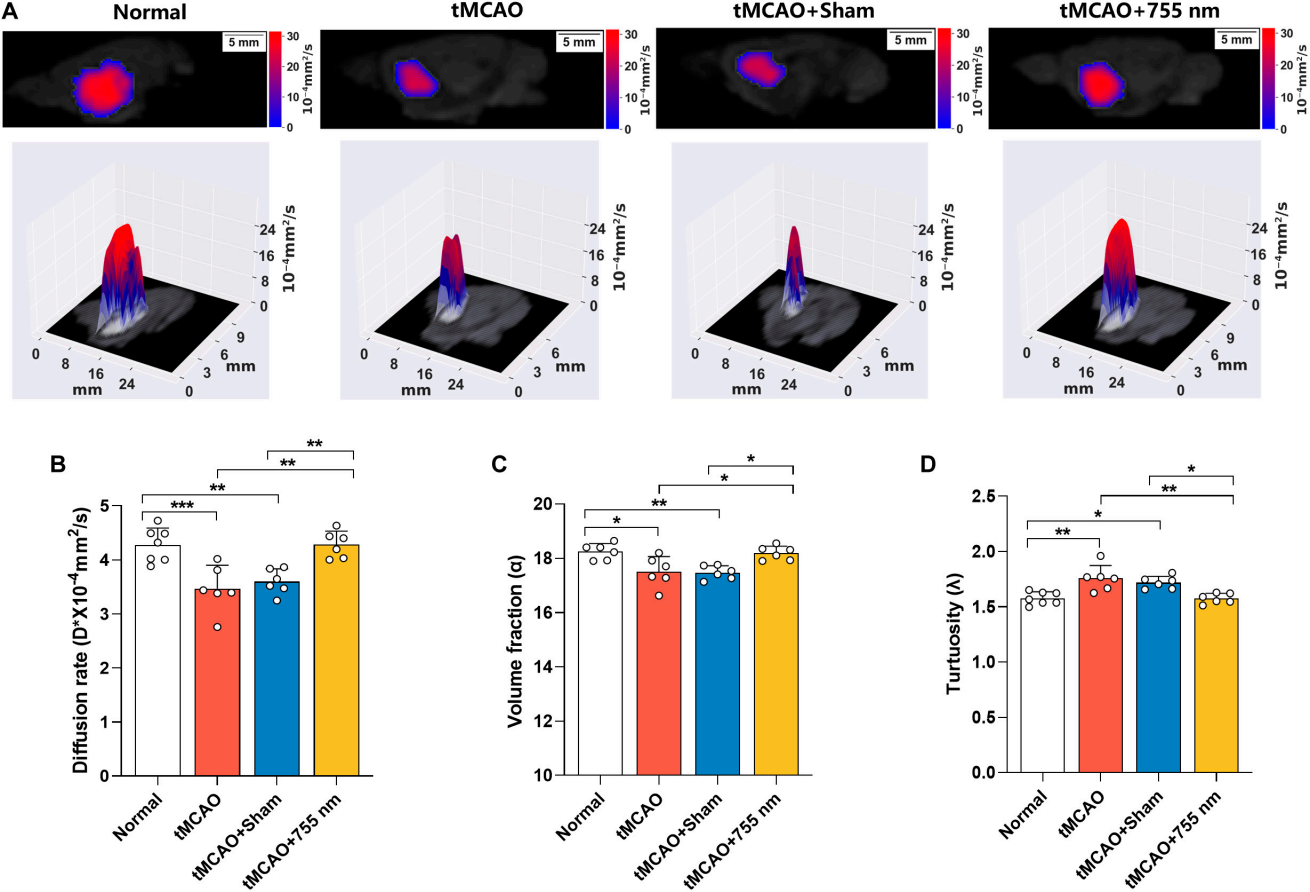
Laser irradiation (755 nm) altered the ECS structure in the brains of tMCAO rats. (A) 2D or 3D D_ECS_-mapping images of the caudate nucleus area in rats of each group and normal controls 7 d after tMCAO surgery. (B to D) Statistical results for ECS structural parameters in the caudate nucleus area: diffusion rate (B), volume fraction (C), and tortuosity (D). Data are expressed as mean ± SEM. *P < 0.05, **P < 0.01, ***P < 0.001, *n* = 6 to 7.

The brain ECS serves as the primary site for the accumulation of pro-inflammatory cytokines and pathological proteins, whereas the ISF drainage is a critical pathway for the clearance of these neurotoxic biomolecules. Tracer-based MRI was applied to assess the effects of 755-nm light irradiation on ISF drainage in the caudate nucleus and superficial cortex of tMCAO rats. The trace revealed that brain ISF flowed from the caudate nucleus to the ipsilateral cortex (Fig. 6A and Fig. S3). Compared to both the tMCAO and tMCAO + Sham groups, ISF drainage was significantly accelerated in the tMCAO + 755 nm group, as evidenced by a shortened half-life (*P* < 0.01, *P* < 0.01 versus tMCAO, tMCAO + Sham) (Fig. 6B). Pearson correlation analysis showed a positive correlation between the half-life of ISF outflow and the load of tau, p-tau, and Aβ in the peri-infarct area (Fig. 6C to E), as well as the deposition of pro-inflammatory cytokines (Fig. 6F to H). These findings suggest that accelerated ISF drainage contributes to the clearance of pathobiomolecules within the brain. The application of D_ECS_-mapping technology showed alterations in the structure and function of the ECS (Fig. 7A). Compared to both the tMCAO and tMCAO + Sham groups, the tMCAO + 755 nm group exhibited an increased local diffusion coefficient (*P* < 0.01, *P* < 0.01 versus tMCAO, tMCAO + Sham) (Fig. 7B), higher volume fraction (*P* < 0.05, *P* < 0.05 versus tMCAO, tMCAO + Sham) (Fig. 7C), and decreased tortuosity (*P* < 0.01, *P* < 0.05 versus tMCAO, tMCAO + Sham) (Fig. 7D), indicating improved ECS environment.

**Fig. 8.**
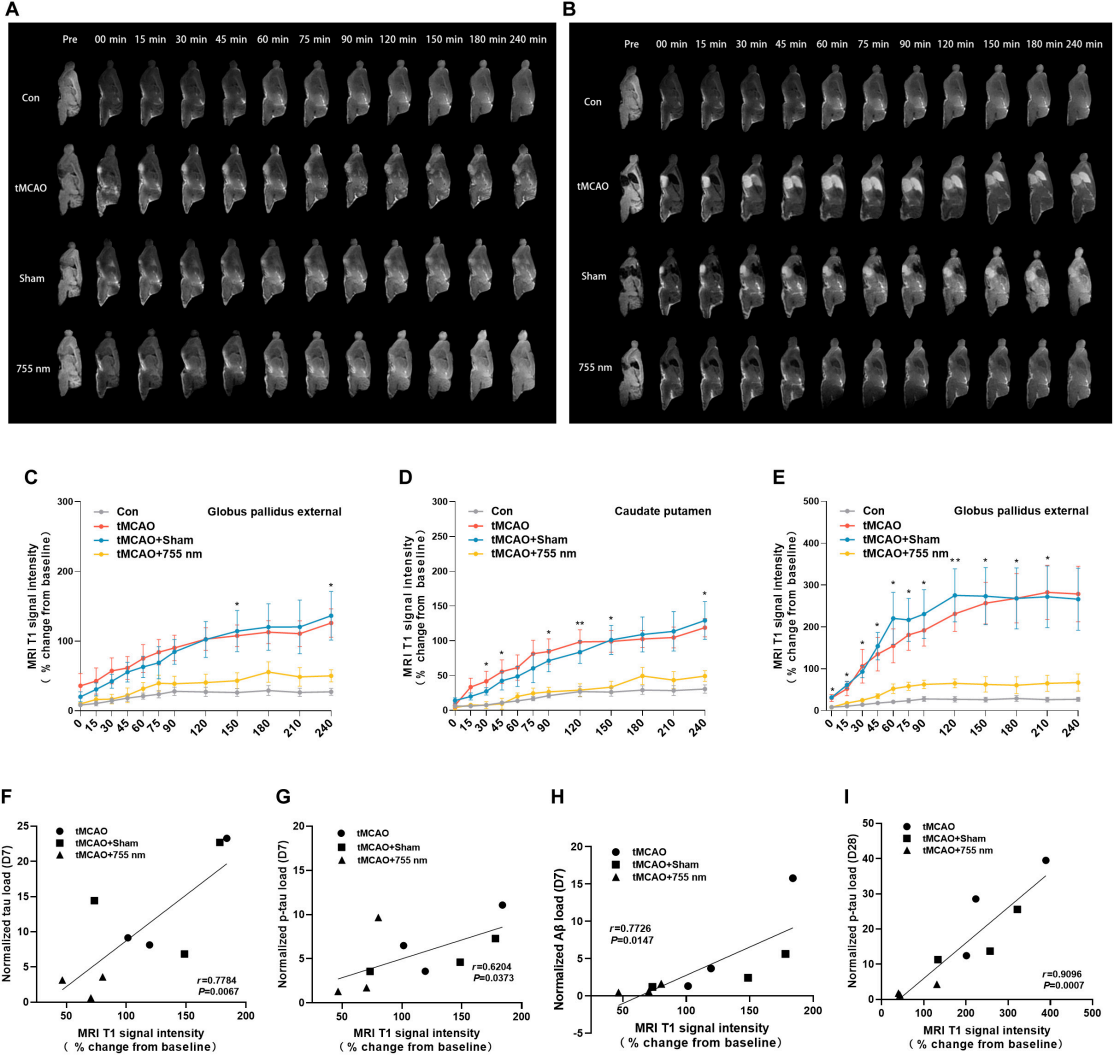
Laser irradiation (755 nm) slowed the influx of CSF-ISF. (A and B) Representative MRI sagittal images of rats in each group and contralateral brains before tracer injection and at 0, 15, 30, 45, 60, 75, 90, 120, 150, 180, and 240 min after tracer injection into the cisterna magna 7 d (A) and 28 d (B) after tMCAO, showing the infiltration of contrast agent into the brain parenchyma. (C to E) MRI T1 signal intensity versus time data obtained from the globus pallidus external (C), caudate putamen (D), and globus pallidus external (E) regions 7 and 28 d after tMCAO. Data are expressed as mean ± SEM. *P < 0.05 or **P < 0.01, *n* = 6. (F to H) Pearson correlation analysis of tau, p-tau, and Aβ load in the peri-infarct region of rats 7 d after tMCAO with normalized MRI T1 signal intensity at 240 min. (I) Pearson correlation analysis of p-tau load in the peri-infarct region of rats 28 d after tMCAO with normalized MRI T1 signal intensity at 240 min.

We injected tracers into the cisterna magna on days 7 and 28 post-stroke to evaluate CSF-ISF inflow in the glymphatic system (Fig. 8A and B). Compared with the Normal group, the tMCAO group and the tMCAO + Sham group showed a significant increase in CSF inflow in the globus pallidus external and the caudate putamen on day 7 (Fig. 8C and D) and day 28 (Fig. 8E), accompanied by notable tracer accumulation. In contrast, the tMCAO + 755 nm group showed a reduction in tracer deposition in the caudate nucleus compared to the tMCAO group. This indicates that tMCAO increased CSF-ISF inflow while slowing outflow. The 755-nm laser effectively mitigated this damage, consistent with results from magnetic tracing studies above. Pearson correlation analysis revealed that tracer deposition on day 7 was positively correlated with tau, p-tau, and Aβ protein levels in the peri-infarct area (Fig. 8F to H), and tracer deposition on day 28 was positively correlated with p-tau levels in the same region (Fig. 8I). These findings indicate that the increased inflow and slowed outflow of CSF-ISF were associated with pathobiomolecule deposition in the brain.

### The 755-nm laser alleviates astrocyte activation after ischemic stroke and altered the expression of AQP4

**Fig. 9.**
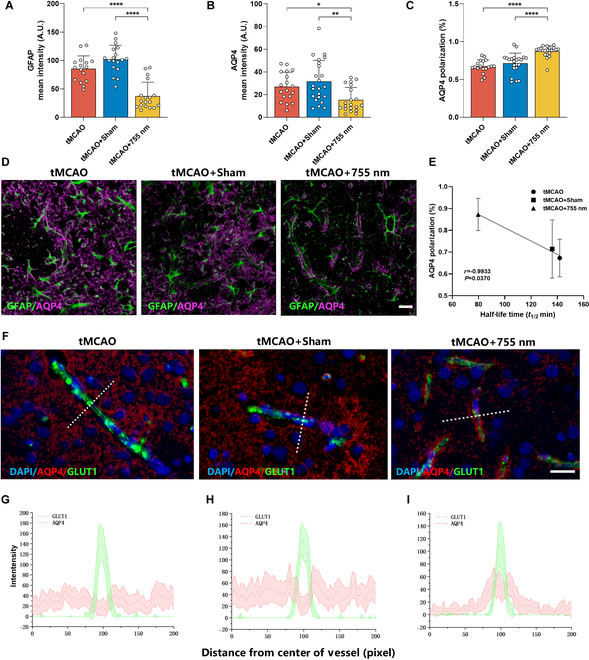
Laser irradiation (755 nm) reduced astrocyte activation and alters AQP4 expression after ischemic stroke. (A) Average fluorescence intensity of astrocytes (GFAP) in the peri-infarct region. (B) Average fluorescence intensity of AQP4 in the peri-infarct region. (C) Quantitative evidence of increased AQP4 polarization in the peri-infarct region of rats in the 755-nm light group compared to the tMCAO group. This is further illustrated in AQP4- and GFAP-stained sections (D), indicating an increase in end-foot AQP4 vascular coverage in the peri-infarct region of the rat brain in the 755-nm treatment group compared to the tMCAO and Sham groups. Scale bar, 20 μm. *n* = 3 rats per group, with 6 random fields of view analyzed per rat in the peri-infarct zone. Data are expressed as mean ± SEM. *P < 0.05, **P < 0.01, ***P < 0.001, or ****P < 0.0001. (E) Pearson correlation analysis of AQP4 polarization degree in the peri-infarct region of rats 7 d after tMCAO with the half-life (*T*_1/2_) of the magnetic tracer experiment. (F to I) Representative immunofluorescence images of AQP4 and GLUT1 in the peri-infarct region of rats in each group (scale bar, 20 μm) (F), illustrating the placement perpendicular to the vessel of a 200-pixel line used to quantify cross-vessel expression, showing an increase in AQP4 expression around vessels in the peri-infarct region of the rat brain in the 755-nm treatment group (G to I). *n* = 2 to 3 rats per group, with 6 to 8 vessels randomly selected for analysis per rat in the peri-infarct zone. Data are expressed as mean ± SEM.

Gliocyte proliferation is a critical pathological response in the peri-infarct region following ischemic stroke. Reactive astrogliosis post-stroke evaluations revealed that stroke induces reactive proliferation of astrocytes. Compared to the tMCAO and tMCAO + Sham groups, 755-nm laser irradiation effectively attenuated astrocyte proliferation around the infarction 7 d post-stroke (*P* < 0.0001, *P* < 0.0001 versus tMCAO, tMCAO + Sham) (Fig. 9A and Fig. S4).

An important link in the CSF-ISF exchange pathway is the water channel protein AQP4, which is predominantly polarized and distributed in the foot processes of astrocytes adjacent to blood vessels. After ischemic stroke, AQP4 polarization is compromised. To investigate whether PBM accelerates the clearance of pathobiomolecules by modifying AQP4 polarization and expression after ischemic stroke, we conducted a series of analyses. Consistent with the MRI observations, on day 7 post-stroke, the brain ISF influx in the tMCAO + 755 nm group was reduced compared to the tMCAO and tMCAO + Sham groups, likely due to the suppression of excessive AQP4 expression post-stroke (*P* < 0.05, *P* < 0.01 versus tMCAO, tMCAO + Sham) (Fig. 9B). Conversely, ISF outflow was significantly accelerated, and tracer deposition was reduced, which was associated with enhanced AQP4 polarization (*P* < 0.0001, *P* < 0.0001 versus tMCAO, tMCAO + Sham) (Fig. 9C and E). Dual immunofluorescence staining for AQP4 and GFAP showed a more pronounced perivascular localization, indicating that the tMCAO + 755 nm group had greater AQP4 coverage at the vascular end feet than the tMCAO and tMCAO + Sham groups (Fig. 9D). Analysis of vascular cross-sections in the peri-infarct area also revealed impaired AQP4 expression in the perivascular space. However, 755-nm laser treatment markedly reversed this impairment, transforming AQP4 expression from a parenchymal type to a perivascular type (Fig. 9F to I). In addition, this effect appears to be sustained, suggesting that 755-nm light exposure may create a continuous positive feedback loop, thereby accelerating ISF drainage and enhancing clearance of pathobiomolecules in the ECS.

## Discussion

In this study, we utilized the parameter-adjustable PBM therapy apparatus to screen for the optimal PBM parameters for treating ischemic stroke and preventing PSCI. We demonstrated that PBM facilitates its effects through the acceleration of molecular transport in the brain ECS. Our results show that among various wavelengths, 755 nm was the most effective in reducing infarct volume and mitigating tissue and functional damage. The 755-nm laser can restore substance transport in the brain ECS by enhancing AQP4 polarization. This improvement facilitates the clearance of pro-inflammatory cytokines and extracellular pathological proteins, ultimately alleviating ischemia–reperfusion injury and preventing cognitive impairment following stroke. These findings offer novel potential therapeutic strategies that leverage the regulation of ISF drainage to promote functional recovery in patients with ischemic stroke. Therefore, 755-nm phototherapy may be a practical and promising strategy for treating ischemic stroke.

Research indicates that above 50% of clinical cases of ischemic stroke primarily present with infarction in the territory supplied by the middle cerebral artery [37]. In recent years, advancements in thrombolytic therapy have substantially improved recanalization rates in stroke patients [1]. Consequently, ischemia–reperfusion injury has become a focal point of research [20]. The tMCAO model induced by the intraluminal filament technique can achieve ischemia–reperfusion by removing the filament [37]. Compared to transcranial occlusion, embolic occlusion, endothelin-1 occlusion, and photothrombosis models, the tMCAO model is widely used in ischemic stroke research due to several advantages: It (a) does not necessitate craniotomy, (b) induces minimal tissue damage, (c) enables precise control over ischemia duration, (d) ensures a stable infarct location, (e) demonstrates relatively consistent severity of brain ischemic injury, and (f) offers high reproducibility [37]. Therefore, this study employs the tMCAO model to mimic the pathological state of cerebral ischemia–reperfusion in patients, aiming to closely reflect clinical scenarios.

Several studies have suggested that PBM therapy may exert positive effects on ischemic stroke [14,15,38]. The optimization of light parameters is a critical factor in achieving effective therapeutic outcomes. The parameter-adjustable PBM therapy apparatus effectively facilitates experimental protocols in the study of ischemic stroke. In this study, to identify the most effect wavelength for treatment, light at wavelengths of 638, 755, and 808 nm were used to treat tMCAO rats. Notably, in this study, the 755-nm laser exposure resulted in the most marked improvement in both tissue and functional outcomes following ischemic injury. The 638-nm laser irradiation also demonstrated functional benefits. Research has shown that 630-nm laser substantially inhibits microglia activation, thereby preventing the inflammatory response after acute ischemic stroke [14]. Light-emitting diode (LED)-red light at 630 nm can improve formaldehyde dehydrogenase (FDH) activity, which led to formaldehyde (FA) degradation, and smash Aβ deposition in the ECS [27]. It reversed memory deficits in the AD animal model by recovering ISF flow [27], and we believe that 638-nm laser could play a similar role in preventing cognitive impairments after stroke. However, the study of ischemic stroke involves a range of complex mechanisms [39]. Moreover, our findings indicate that the 755-nm laser demonstrates more pronounced therapeutic effects than the 638-nm laser in certain experimental outcomes. For example, improvements in mNSS scores were observed in the tMCAO + 755 nm group starting on day 7, whereas such improvements were not evident in the tMCAO + 638 nm group until day 14. Additionally, histological analyses (H&E, TUNEL, and Nissl staining) revealed significantly lower cell survival rates in the tMCAO + 638 nm group compared to the tMCAO + 755 nm group. Therefore, it is suggested that the mechanism by which 755-nm laser irradiation reduces ischemia–reperfusion injury and prevents PSCI may differ from that of 638 nm. The 755-nm laser irradiation warrants further experimental investigation. Conversely, the 808-nm laser exhibited detrimental effects. In the tMCAO + 808 nm group, survival rates were significantly reduced compared to the control group. A substantial number of rats died during the 7-d light treatment period. However, the recorded body weights and mNSS scores of the tMCAO + 808 nm group were notably better than those of the control group. This improvement is likely not attributable to the effectiveness of the treatment, but rather to the possibility that 808-nm laser irradiation may have exacerbated ischemic brain injury, which resulted in only tMCAO rats with initially mild infarcts surviving. Similarly, since the surviving rats in the tMCAO + 808 nm group presented with smaller infarct volumes on day 1, the smaller infarct volumes observed on day 28 do not represent a significant reduction in infarct size. The results of H&E, TUNEL, and Nissl staining in the Supplementary Materials are consistent with our findings. Literature has reported that 810-nm laser irradiation can induce local temperature increases, potentially leading to thermal injuries or other adverse effects, which raises safety concerns [27]. This is a key reason why we decided not to proceed with the tMCAO + 808 nm group in subsequent experiments. The primary goal in ischemic stroke treatment is the rapid restoration of blood flow [40], yet reperfusion can paradoxically exacerbate brain injury, primarily due to the generation of ROS during reoxygenation [41]. Mitochondria, a source of post-ischemic ROS, can have their respiration enhanced by 670- and 808-nm lasers, which stimulate cytochrome c oxidase (COX) activity [42]. It is known that enhancing mitochondrial function early during reperfusion may paradoxically exacerbate tissue damage. In contrast, applying specific inhibitory NIR wavelengths, such as 750 and 950 nm, reduces COX activity and decreases superoxide generation [43]. The use of inhibitory NIR light during reperfusion has shown neuroprotective effects in rat models of ischemic stroke [20]. These findings align with our experimental results and lead us to utilize a 755-nm laser in subsequent experiments to explore its mechanisms of action and clinical translational potential for ischemic brain injury. Additionally, in studies where the therapeutic wavelength was selected, we observed an increase in body weight in the treatment group, which may result from several contributing factors [44]. The treatment can enhance the overall health and vitality of the tMCAO rats. Enhanced survival status is frequently associated with increased appetite and food intake, both of which can lead to weight gain [45]. The tMCAO rat model is characterized by chronic inflammation and persistent stress [46]. Effective treatment can alleviate these detrimental effects, thereby reducing energy expenditure and promoting weight gain.

In this study, we demonstrated that 755-nm laser markedly accelerated the brain ISF drainage in tMCAO rats, effectively reducing the aggregation and deposition of tracers. Our application of D_ECS_-mapping technology revealed that 755-nm laser altered the microscopic structural parameters of the brain ECS, including tortuosity, volume fraction, and local diffusion rate. These findings align with recent studies that PBM reduces the tortuosity of the brain ECS and accelerates the diffusion of substances within this space, thereby promoting ISF drainage [26]. In addition, transcranial optogenetics have been used to generate synthesized ionic waves in the brain ECS, enhancing the perfusion of CSF into the brain parenchyma ISF [25]. This has been adopted to AD pathology, where various wavelengths of PBM have shown to disrupt Aβ deposition in the ECS and restore ISF drainage [17,27]. In summary, PBM promotes ISF drainage under both physiological and pathological conditions, with effects varying depending on the light exposure parameters and the state of the brain tissue.

Within minutes of an ischemic stroke, microglia become overactivated, releasing large amounts of pro-inflammatory cytokines such as IL-1β, IL-6, and TNF-α [47]. This release triggers a cascade that exacerbates brain damage. Under ischemic conditions, impaired drainage of brain ISF results in the accumulation of these cytokines in the ECS, further aggravating inflammation and ischemic injury. Consequently, promoting the clearance of pro-inflammatory cytokines enhances functional recovery after stroke [48]. Our previous research indicated that m-EAI (modified epidural arterial implantation) reduced the accumulation of IL-1β, IL-6, and TNF-α by accelerating ISF drainage and improved the hyperactivation of microglial and astrocyte cells on day 3 after tMCAO [7]. The effectiveness of m-EAI depends on the patient’s vascular condition. Arteries with atherosclerosis pulsate weakly, and the degree of arterial pulsation varies among individuals, affecting treatment outcomes [49]. In contrast, PBM is not limited by the patient’s vascular status and can be more easily administered in a controlled manner, serving as a more robust and generalizable treatment option for ischemic stroke [50]. In our study, 755-nm laser significantly decreased the levels of pro-inflammatory cytokines in tMCAO rats on day 7 after stroke, correlating with accelerated ISF drainage. This reduction in pro-inflammatory cytokines inhibited the activation of microglia in the penumbra region following ischemic attack, further alleviating brain ischemia–reperfusion injury. Following 755-nm laser irradiation, microglial cells exhibited increased branching, which may indicate a shift from an activated to a more quiescent state. This morphological change resulted in a more dispersed appearance of these cells under the microscope [17]. As a result, the nuclear density per unit area appeared lower. This shift reflects the modulation of microglial cell activity by 755-nm laser treatment, which likely contributes to the attenuation of inflammatory responses and supports neuroprotection and tissue repair processes. Additionally, we speculate that the microstructural changes in the brain ECS induced by 755-nm laser therapy are related to alleviation of inflammatory response, slight cell swelling, and a relative reduction of smaller, tightly arranged microglial cells.

More than one-third of stroke patients may develop PSCI. Current studies have demonstrated that neurodegenerative changes after ischemic stroke, such as accumulation of Aβ plaques in the brain parenchyma and tau tangles in nerve fibers, play an important role in the occurrence and development of PSCI [5,6]. Under normal conditions, tau protein is predominantly located in neuronal axons, where it plays a key role in stabilizing microtubules [51]. However, following ischemic injury, tau protein can dissociate from the cytoskeleton and undergo abnormal phosphorylation, resulting in the formation of p-tau [52]. This modification can contribute to the development of neurofibrillary tangles and neuronal dysfunction. In the early stages of stroke, particularly within the first 7 d, p-tau levels can significantly increase [52]. This elevation is attributed to the activation of multiple kinases by ischemia-induced cellular stress, which promotes excessive tau phosphorylation [52]. While Aβ is primarily associated with AD, emerging evidence suggests that Aβ deposition may also occur within 7 d following ischemic stroke [53]. Ischemic stroke triggers accelerated deposition of Aβ, yet the candidate proteins associated with their generation and degradation remain unchanged, likely due to interference in the clearance pathways [53]. Pathophysiological mechanisms related to PSCI uncovers potential therapeutic targets. Our study shows that 755-nm laser alleviates the deposition of endogenous neuropathological proteins after ischemic stroke and reduce phosphorylated tau and Aβ plaques, thereby improving cognitive function and preventing PSCI. Therefore, PBM restores ISF drainage, thereby accelerating the clearance of neuropathological substrates and reducing their deposition, which establishes the functional basis for PBM to prevent PSCI. Collectively, the brain ECS and ISF is the anatomy of brainwashing, which can remove metabolic waste and toxins from tissues [13]. The acceleration of substance transport in the brain ECS promotes the clearance of pathobiomolecules, including pro-inflammatory cytokines and neuropathological proteins, thereby alleviating both inflammatory responses and pathological protein deposition.

**Fig. 10.**
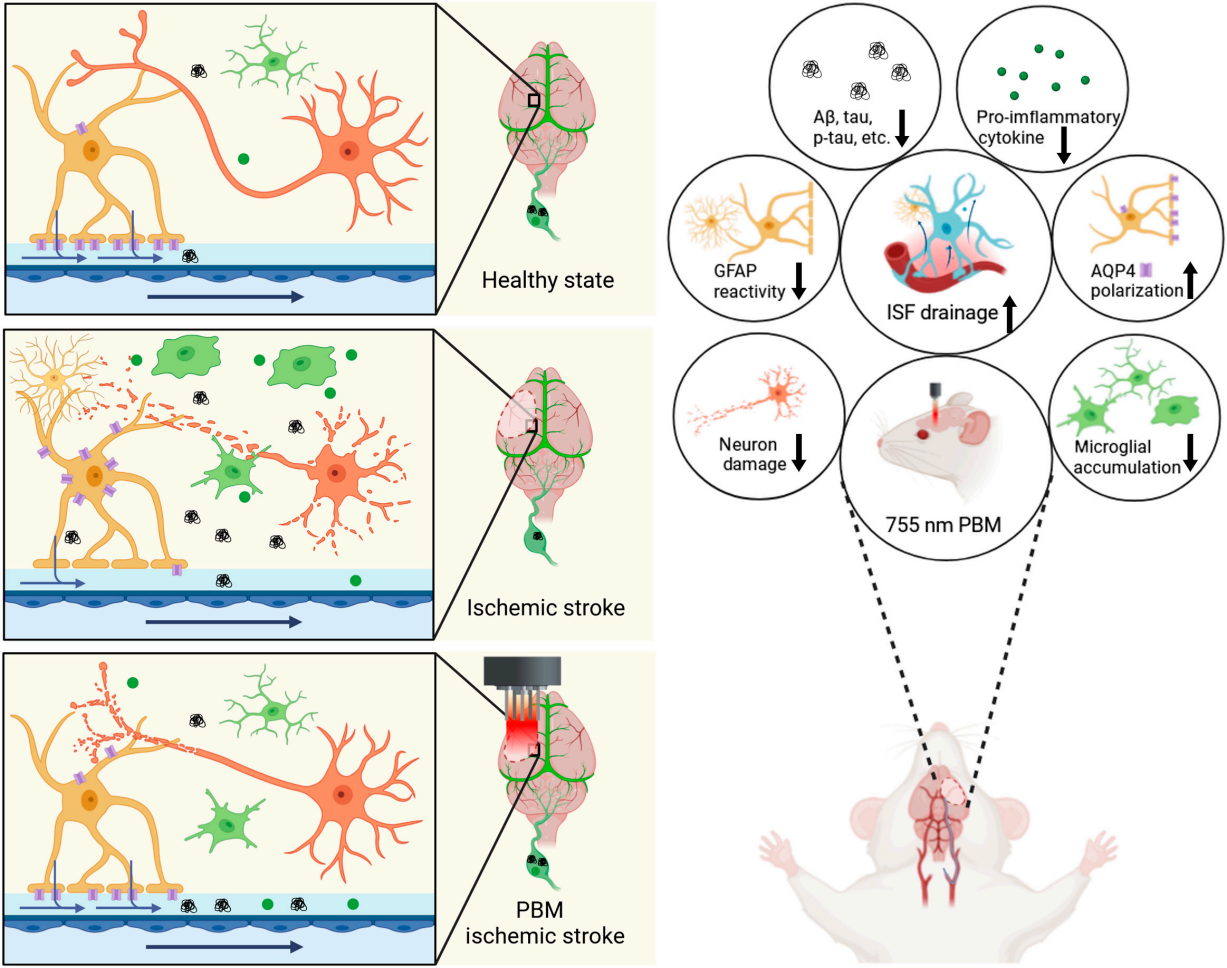
Schematic representation of treatment of ischemic stroke with PBM in 755 nm. The right image is an annotation to the left. Following ischemic stroke, various cell types within the central nervous system undergo distinct morphological changes. In the peri-infarct region, neurons exhibit marked alterations, characterized by the loss of cell bodies and axons. Glial cells, in response to injury, transition into an “activated state”, producing pro-inflammatory cytokines. Activated astrocytes lead to the loss of the polarized characteristics of AQP4 distribution, i.e., the previously concentrated AQP4 distribution in specific areas becomes nonuniform or redistributed. Brain ISF drainage slows down, and pathological proteins and pro-inflammatory cytokines are deposited in the brain ECS. The 755-nm light reverses these phenomena. This is a schematic figure for identification purposes and is not be scaled. This is licensed from BioRender.

In addition, ischemic stroke triggers local inflammatory response, activating astrocytes that affect AQP4 polarization [54]. Under physiological conditions, AQP4 is primarily polarly distributed at the perivascular end feet of astrocytes, playing a crucial role in maintaining intracranial water homeostasis and CSF circulation [55]. However, following ischemic stroke, activated astrocytes lead to the loss of the polarized characteristics of AQP4 distribution [56]. AQP4 is dynamically and rapidly trafficked between intracellular vesicles and the astrocytic plasma membrane [57], and its polarity governs the molecular transportation in the brain ECS [58]. The translocation of AQP4 from intracellular vesicles to the cell surface occurs when a hypoxic (or hypotonic) environment triggers an influx of calcium ions, through transient receptor potential vanilloid 4 (TRPV4) channels, which bind to and activate CaM [57]. Recently, AQP4 subcellular trafficking has been validated as a potential target for edema treatment [59]. This was done through the administration of the Food and Drug Administration (FDA)-licensed trifluoperazine to rats subjected to spinal crush, TBI, and stroke, leading to a reduction in edema and enhanced functional and behavioral recovery [59]. Our findings indicate that 755-nm laser irradiation effectively attenuated astrocyte proliferation around the infarction, and these alterations in cell types at these sites might lead to structural changes in the brain ECS and variations in ISF drainage. The overexpression of AQP4 following ischemic stroke was suppressed, thereby decreasing the CSF-ISF influx and ISF accumulation. Concurrently, AQP4 polarization is enhanced, which accelerates the outflow of brain ISF (Fig. 10). Such changes restrained water accumulation and the clearance obstruction of harmful substances in the brain after ischemic attack, thereby alleviating the inflammatory response and facilitating the recovery of neural functions. Studies have demonstrated that the Rhou and Epha5 genes are involved in the morphological changes of astrocytes in response to both physical and chemical stimuli. Furthermore, Rtn1, Tmcc1, and Arl6ip1 are implicated in the formation of the endoplasmic reticulum, a process that is critical for the synthesis and transport of AQP4 [58]. Murdock et al. [58] demonstrated that 40-Hz multisensory stimulation, including light stimuli, can modulate the expression of these genes. Multisensory stimulation can increase the expression of Lamc1 in pericytes [58]. Lamc1 is a laminin subunit that interacts with dystroglycan, which helps stabilize AQP4 at the astrocyte end feet [60]. Therefore, future studies investigating the mechanisms by which PBM regulates AQP4 polarization may benefit from focusing on these pathways. Moreover, inflammation also directly impacted the expression level and functional activity of AQP4. Pyroptosis, a form of pro-inflammatory cell death, has been identified as a trigger for Aβ accumulation after cerebral ischemia–reperfusion injury through impairing AQP4 polarization [61]. Studies suggest that decreasing pro-inflammatory cytokines, such as IL-6 and IL-1β, can effectively inhibit pyroptosis induced by cerebral ischemia–reperfusion injury [62]. Therefore, this in-depth study of the dynamic changes in AQP4 during an inflammatory response is crucial for understanding the pathogenesis of inflammation-related neurological diseases, providing a theoretical basis for formulating novel treatment strategies. This polarization of AQP4 is closely related to the clearance of neuropathological proteins such as tau and Aβ. A previous study of AD models has shown that impaired glymphatic function due to altered AQP4 polarization leads to compromised tau clearance [63]. Studies have shown that the absence of perivascular AQP4 localization impairs glymphatic exchange in mice and promotes the formation of Aβ plaques [49]. Chronic cerebral hypoperfusion induces dementia after ischemic stroke in rats by interfering with Aβ and tau clearance via the glymphatic pathway, with neuroinflammatory response that changes AQP4 distribution from perivascular to parenchymal [64]. Therefore, by improving AQP4 polarization, PBM restores ISF drainage after stroke, thereby preventing PSCI.

Our study has some limitations and can be further explored in the future. In the present study, we selected the most efficacious wavelength from the 3 currently available options. While 755 nm exhibited superior efficacy in the treatment of ischemic stroke, we acknowledge that there may be other wavelengths or parameter configurations that could potentially offer even better therapeutic outcomes. Currently, no standardized multi-wavelength, customizable parameter PBM apparatus is available on the market [14,65], which is one of the reasons we developed our apparatus. The primary advantage of our apparatus is its integration of multiple standard laser emitters and the ability for users to customize parameters. This design facilitates the screening of the most effective wavelengths for treating ischemic stroke and can also be applied to other diseases that may benefit from PBM therapy. The PBM therapy apparatus used in this study is configured to emit only 3 specific wavelengths and does not support the simultaneous emission of multiple wavelengths. To accommodate the varying needs of different diseases in future applications, it will be necessary to expand the device’s wavelength range based on the unique characteristics of each condition. Additionally, our investigation into the mechanisms of PBM therapy for ischemic stroke has centered on the brain ECS. Ischemic stroke triggers a series of pathological changes, including oxidative stress, metabolic abnormalities, calcium overload, and excitotoxicity [8,39]. These complexities necessitate further research to fully elucidate the mechanism of 755-nm laser therapy for treating ischemic stroke. We believe that other toxic substances, such as reactive ROS and free radicals, accumulated lactate, glutamate, and calcium ions, are involved in the stroke process [8,39]. These substances may also be cleared faster with improved molecular transport in the brain ECS [13]. However, more studies are needed to confirm this idea. While this study primarily presents preclinical data, the findings provide a crucial theoretical foundation and technical framework for the potential application of PBM therapy in human patients. Moving forward, several considerations are essential for translating these findings into clinical practice. These include the need for more rigorously designed randomized controlled trials, particularly focusing on various neurological disorders, to assess the safety, efficacy, and long-term effects of PBM therapy [29]. Furthermore, developing personalized PBM parameters, such as wavelength, power density, and exposure duration, tailored to the disease state, lesion location, and individual patient characteristics, will be key to optimizing therapeutic outcomes and minimizing potential side effects. Transcranial PBM administered within 24 h of stroke onset has demonstrated safety, with no adverse reactions or increased mortality observed [21,24,66]. However, NIR or red light may induce a “thermal effect”, which could result in side effects such as headaches, sleep disturbances, and insomnia, typically associated with temperature elevations [67,68].

In summary, our findings highlight the neuroprotective effects of 755-nm light via the acceleration of ISF drainage during ischemic stroke. This study provides invaluable insight into the mechanisms by which 755-nm light modulates the brain ECS. PBM therapy offers a minimally invasive and effective approach that holds promise for treating ischemic strokes.

## Data Availability

The data are contained within the article and the Supplementary Materials.
