# Supplementary material for: Accelerated Molecular Transportation in the Brain Extracellular Space with 755-nm Light Attenuates Post-Stroke Cognitive Impairment in Rats
Source: Cyborg Bionic Syst. 2025 May 6;6:0262. doi: 10.34133/cbsystems.0262 (PMC12053100; doi:10.34133/cbsystems.0262)
Supplement: Supplementary 1 — Materials and Methods Figs. S1 to S5 Tables S1 to S10 [file cbsystems.0262.f1.zip › Supplementary Material for CBS_0321.docx]

**Supplementary Material for Cyborg and Bionic Systems:**

**MATERIALS AND METHODS**

**Cognitive Behavioral Testing**

*mNSS:* The specific scoring criteria for mNSS are shown in Table S1.

Table S1. The modified Neurological Severity Scores

|  | Points |
| --- | --- |
| Motor tests |  |
| Raising rat by the tail | 3 |
| 1 Flexion of forelimb |  |
| 1 Flexion of hindlimb |  |
| 1 Head moved >10° to vertical axis within 30 s |  |
| Placing rat on the floor (normal=0: maximum=3) | 3 |
| 0 Normal walk |  |
| 1 Inability to walk straight |  |
| 2 Circling toward the paretic side |  |
| 3 Fall down to the paretic side |  |
| Sensory tests | 2 |
| 1 Placing test (visual and tactile test) |  |
| 2 Proprioceptive test (deep sensation, pushing the paw against the table edge to stimulate limb muscles) |  |
| Beam balance tests (normal=0; maximum=6) | 6 |
| 0 Balances with steady posture |  |
| 1 Grasps side of beam |  |
| 2 Hugs the beam and one limb falls down from the beam |  |
| 3 Hugs the beam and two limbs fall down from the beam, or spins on beam (>60 s) |  |
| 4 Attempts to balance on the beam but falls off (>40 s) |  |
| 5 Attempts to balance on the beam but falls off (>20 s) |  |
| 6 Falls off: No attempt to balance or hang on to the beam (<20 s) |  |
| Reflexes absent and abnormal movements | 4 |
| 1 Pinna reflex (head shake when touching the auditory meatus) |  |
| 1 Corneal reflex (eye blink when lightly touching the comea with cotton) |  |
| 1 Startle reflex (motor response to a brief noise from snapping a clipboardpaper) |  |
| 1 Seizures, myoclonus, myodystony |  |
| Maximum points | 18 |

**Pathological Staining**

After obtaining the brain, it was fixed in paraformaldehyde for 24 hours, embedded in paraffin, and sectioned into slices of 6 μm thickness using a paraffin microtome.

*HE Staining:* The brain tissue sections were placed in xylene for dewaxing, followed by hydration in alcohol at various concentrations; The hydrated brain tissue sections were stained in hematoxylin solution for 3 minutes, followed by differentiation in a differentiating solution, rinsed under running water, and then dehydrated in a gradient of alcohol for 5 minutes each. After dehydration, they were placed in eosin dye for 5 minutes, again dehydrated and clarified in alcohol and xylene, mounted with neutral gum, and properly labeled. Finally, the sections were observed under a microscope, and images were captured and analyzed.

*Nissl Staining:* Referencing the HE staining procedure, start by deparaffinizing the paraffin sections to water. Place the hydrated tissue sections in Nissl stain for 10 minutes, rinse with running water, differentiate with 0.1% glacial acetic acid, terminate the reaction with water washing. Control the degree of differentiation under the microscope. After water washing, place the tissue sections in an oven to dry, then put them in clean xylene for 10 minutes for clearing, and mount them with neutral gum; finally, capture and analyze images under the microscope.

*TUNEL Staining:* Start by deparaffinizing the paraffin sections to water; Add protease K without DNase to the brain tissue and incubate at 37°C for 20 minutes, followed by three PBS washes; Then, add permeabilization working solution over the brain tissue and incubate at room temperature for 20 minutes, followed by three PBS washes; Place the sections in a 3% H2O2 solution and incubate in the dark at room temperature for 20 minutes, followed by three PBS washes; Mix Recombinant TdT enzyme, Biotin-dUTP Labeling Mix, and Equilibration Buffer at a ratio of 1 µL:5 µL:50 µL, drop it onto the brain tissue, place the brain tissue sections in a humidified chamber and incubate in the dark at 37°C for 1 hour. Add a small amount of water in the humidified chamber to maintain humidity, and wash again three times with PBS; Subsequently, add the sections to a mixture of Streptavidin-HRP and TBST (ratio 1:200), continue to incubate in the dark at 37°C for 30 minutes, and wash three times with PBS; Drop the freshly prepared DAB color development solution onto the sections, observe the color development under the microscope. If the cell nuclei appear brown-yellow, it indicates positivity, and rinse the sections with pure water to terminate the color development; Counterstain with hematoxylin for 1 minute, wash with pure water, counterstain with ammonia to turn blue, and wash again with pure water; Finally, dehydrate the sections multiple times with 100% alcohol, soak in n-butanol for 5 minutes, place them in xylene for clarification, air dry, and mount the slides.

**RESULTS**

**Comparison of the effects of different PBMs on reducing neuronal damage in tMCAO rats**

In order to further clarify the protection of different wavelength light exposure on tMCAO rats, HE, Nissl, TUNEL and NeuN staining were used to evaluate neuronal damage and survival on day 7 after tMCAO (Fig. S1,S2A-C). As shown in Fig. S1, HE staining revealed that normal cells (arrows in Fig. S1 column 1) had clear outlines, while numerous cell necrosis (triangles in Fig. S1 column 1, S2A) occurred in the tMCAO group and tMCAO+Sham group. The proportion of necrotic cells in the tMCAO+755 nm group was significantly reduced. Compared to the tMCAO group and the tMCAO+Sham group, the percentage of surviving cells in the tMCAO+638 nm group was significantly increased. However, it was significantly lower when compared to the tMCAO+755 nm group. TUNEL staining showed typical DNA fragmentation (Fig. S1 column 2, S2B). A large number of TUNEL+ cells were present in the tMCAO and tMCAO+Sham groups. The percentage of TUNEL+ cells in the tMCAO+638nm group and the tMCAO+755 nm group was significantly lower compared to both the tMCAO group and the tMCAO+Sham group. Nevertheless, this number was markedly higher in the tMCAO+638 nm group relative to the tMCAO+755 nm group. This indicates that 755 nm laser effectively alleviates cell death in tMCAO rats. While 638 nm laser irradiation demonstrated a beneficial effect in reducing cell death, its impact was less significant compared to the therapeutic effects observed with 755 nm laser irradiation.

We also used Nissl staining (Fig. S1 column 3, S2C) to examine neuronal damage in tMCAO rats. It was observed that the tMCAO and tMCAO+Sham group had a reduced number of intact neurons. The neurons had irregular shapes and disordered arrangement. However, neuronal damage in the corresponding brain regions was significantly reduced in the tMCAO+755 nm group. The findings obtained with 638 nm laser irradiation are consistent with the observations mentioned previously. The results obtained from the 808 nm laser treatment group may be influenced by the overall survival rate of the rats. The data in HE, Nissl, TUNEL indicate a notable improvement in cell survival within this group. We collected brain sections from different groups for immunofluorescence staining of the neuronal marker NeuN(Fig. S2D-F). The results showed that the number of surviving neurons in the tMCAO+755 nm group was significantly higher compared to the tMCAO and tMCAO+Sham group. These findings suggest that 755 nm laser can substantially reduce neuronal damage.


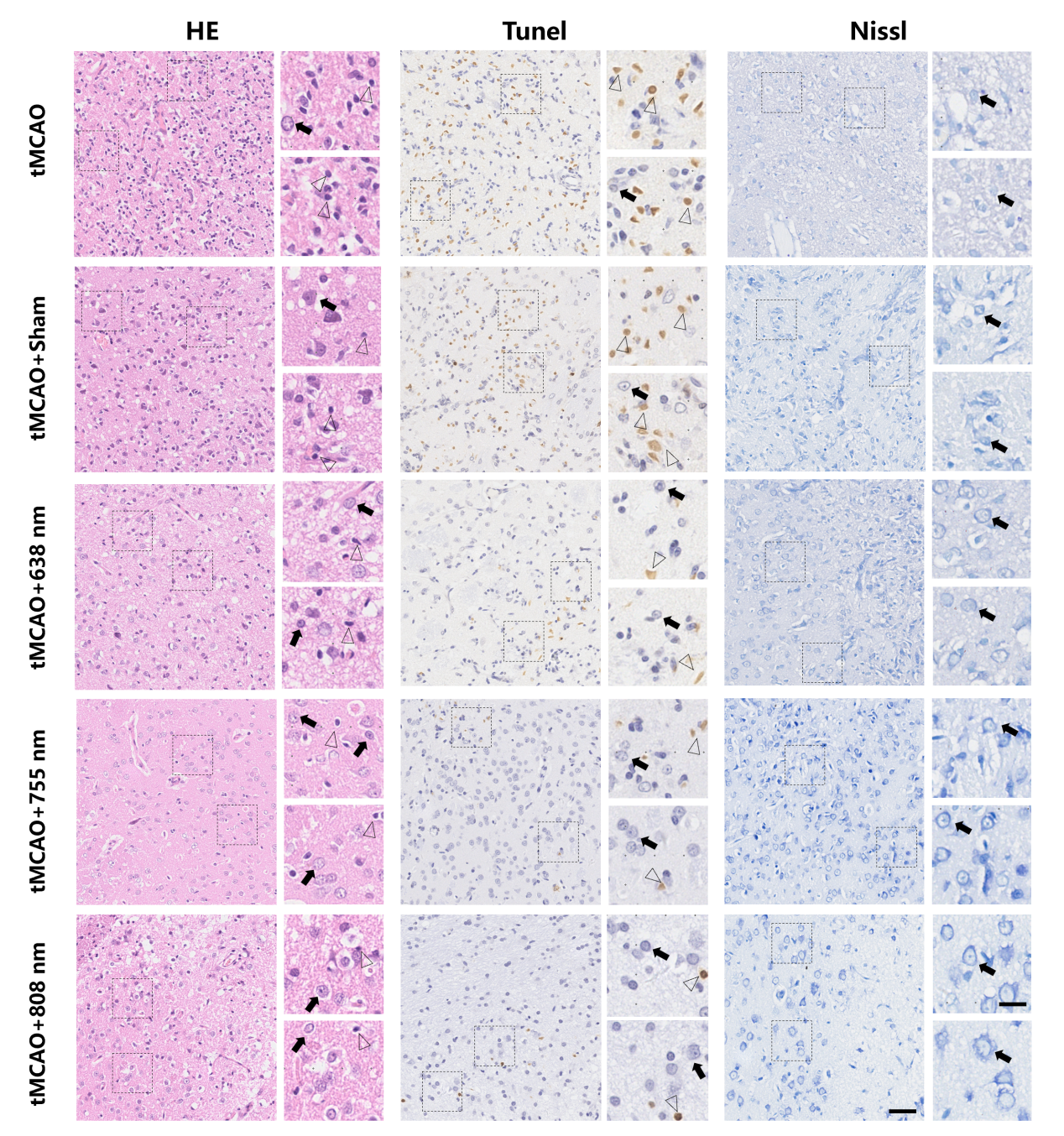
**Fig. S1.** PBMs reduced neuronal damage in tMCAO rats. Representative images of rat brains stained with HE, Nissl, and Tunel for each group (scale bar, 50 μm). Enlarged images show typical cellular morphology (scale bar, 20 μm): HE staining show changes in cellular morphology in the peri-infarct area. Arrows indicate cells with clear contours and dense structure. Triangles represent damaged cells with shrunken cell bodies and condensed nuclei. TUNEL staining shows DNA fragmentation in the peri-infarct area. Arrows indicate intact cells. Triangles represent apoptotic neurons with shrunken and condensed nuclei. Nissl staining shows changes in neurons in the peri-infarct area. Arrows indicate intact neurons.


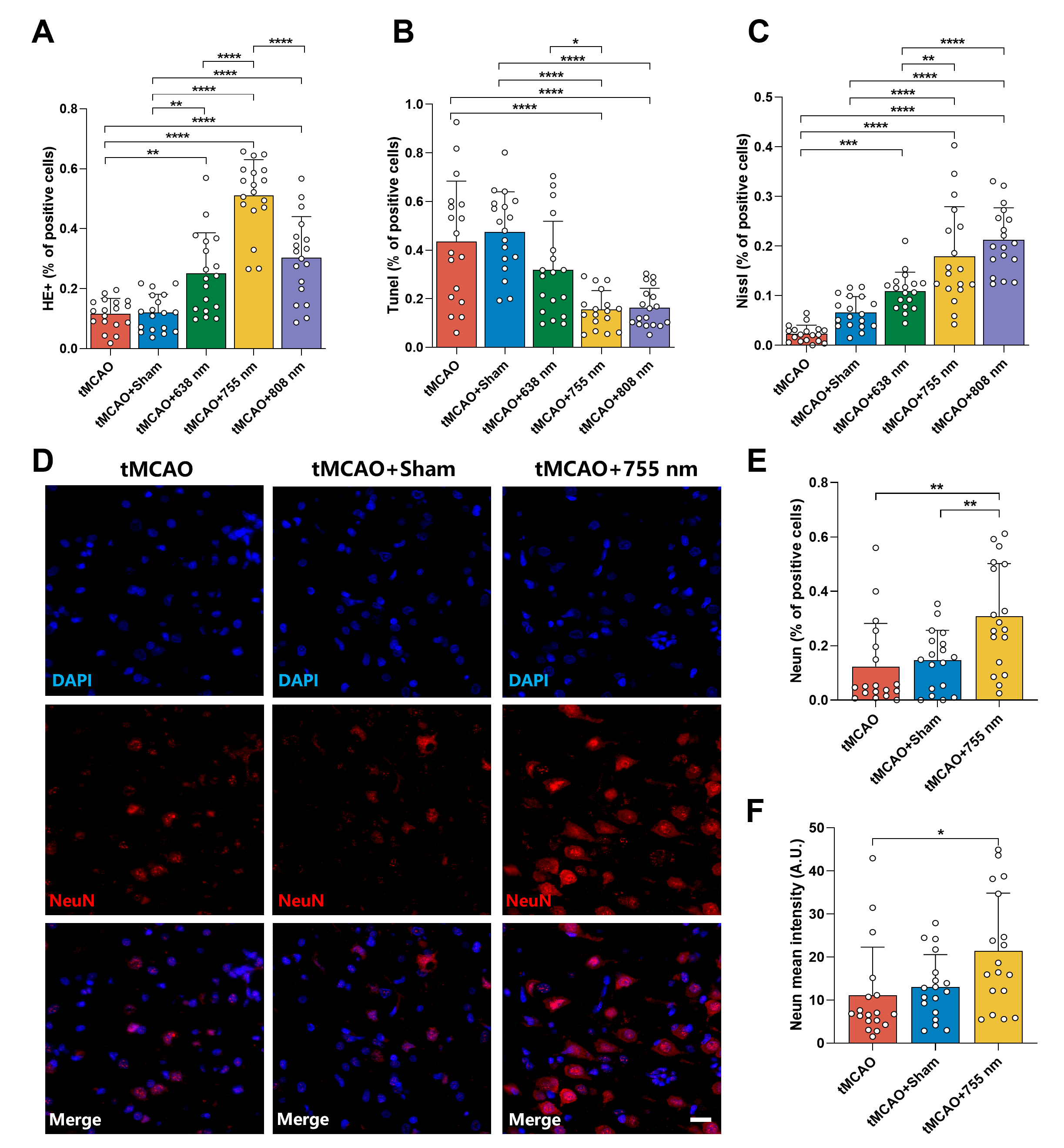


**Fig. S2.** PBMs reduced neuronal damage in tMCAO rats. (A) Percentage of intact cells (HE+) in the peri-infarct area; (B) Percentage of TUNEL+ cells in the peri-infarct area; (C) Percentage of intact neurons (Nissl+) in the peri-infarct area; (D) Representative fluorescent images of NeuN+ neurons in the peri-infarct area 7 days after tMCAO. NeuN+ (red) and DAPI (blue), scale bar = 20 μm; (E) Average fluorescence intensity of viable neurons (NeuN+) in the peri-infarct area; (F) Percentage of viable neurons (NeuN+) in the peri-infarct area. n = 3-4 per group, with 6 random fields of view analyzed per rat in the peri-infarct area. Individual data points are shown as open circles. All data are expressed as mean ± s.e.m., **P* < 0.05, ***P* < 0.01, ****P* < 0.001, or *****P* < 0.0001.


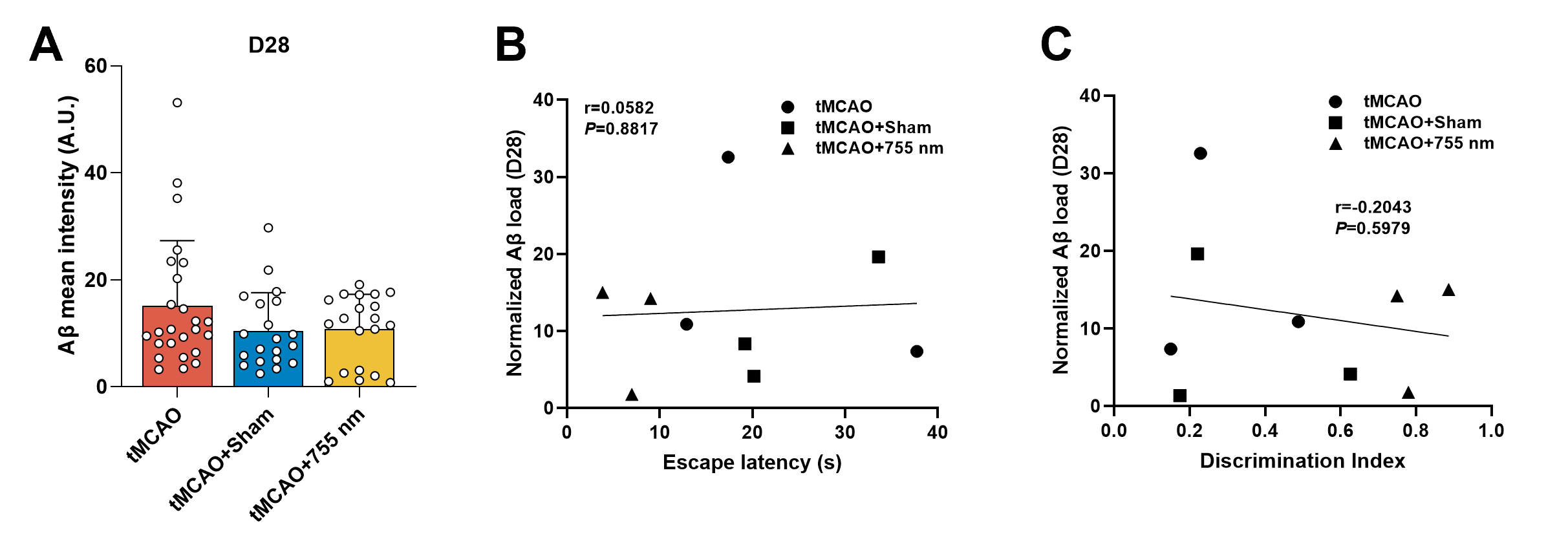


**Fig. S3.** 755 nm laser irradiation reduced the deposition of endogenous tau, p-tau, and Aβ. (A-C) Mean fluorescence intensity of Aβ in the peri-infarct area 28 days after tMCAO; In (A) figure, n = 3 per group, with 6 random fields of view analyzed per rat in the peri-infarct area. Data are expressed as mean ± s.e.m. (B-C) The Pearson correlation coefficient analysis of Aβ load in the peri-infarct area 28 days after tMCAO with the discrimination index in the NOR test (B) and escape latency in the MWM test (C).


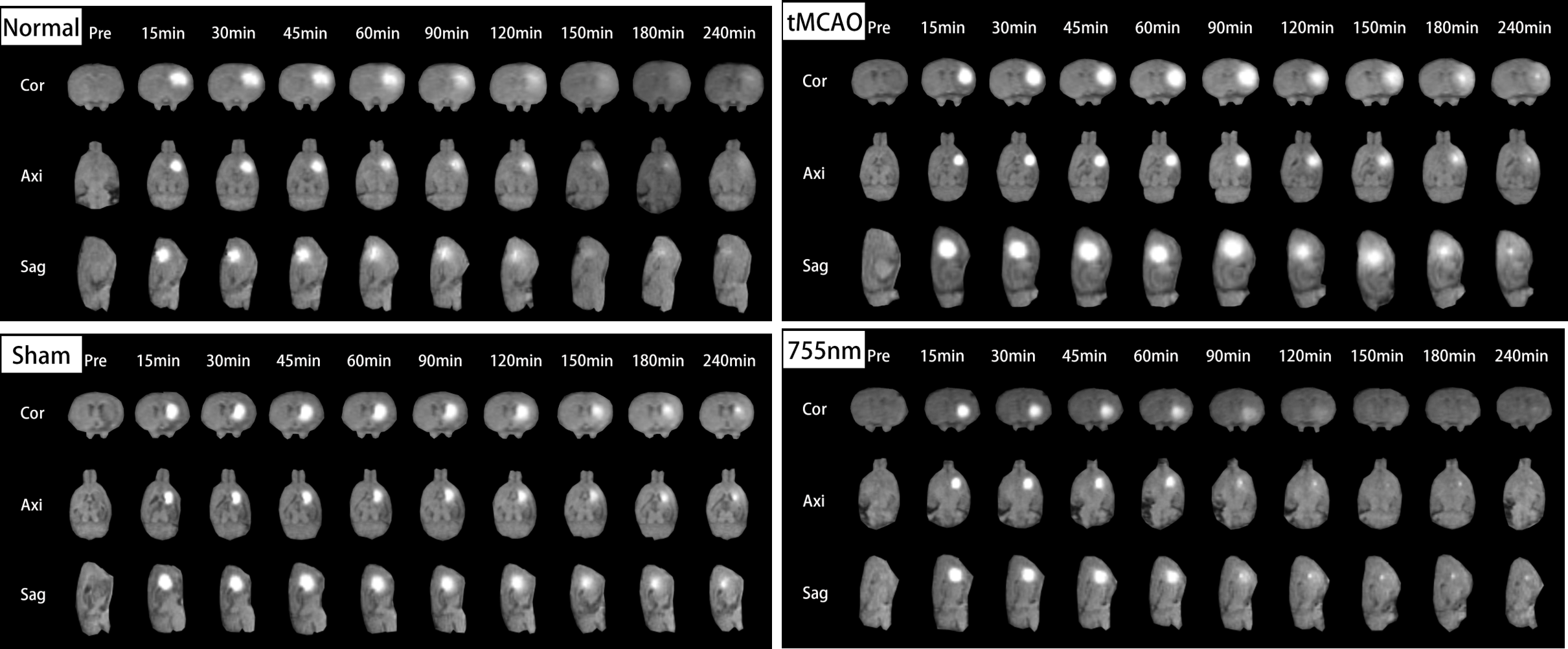


**Fig. S4.** 755 nm laser irradiation accelerated the outflow of ISF in tMCAO rats. Coronal, sagittal and axis MRI images of rats in each group 7 days after tMCAO and normal controls at 15 min, 30 min, 45 min, 60 min, 90 min, 120 min, 150 min, 180 min, and 240 min after tracer injection into the caudate nucleus area. After the tracer is injected into the caudate nucleus of the rats, a circular signal enhancement area becomes visible, which gradually expands over time along with a gradual reduction in signal strength, with differences in clearance rates among the different groups.


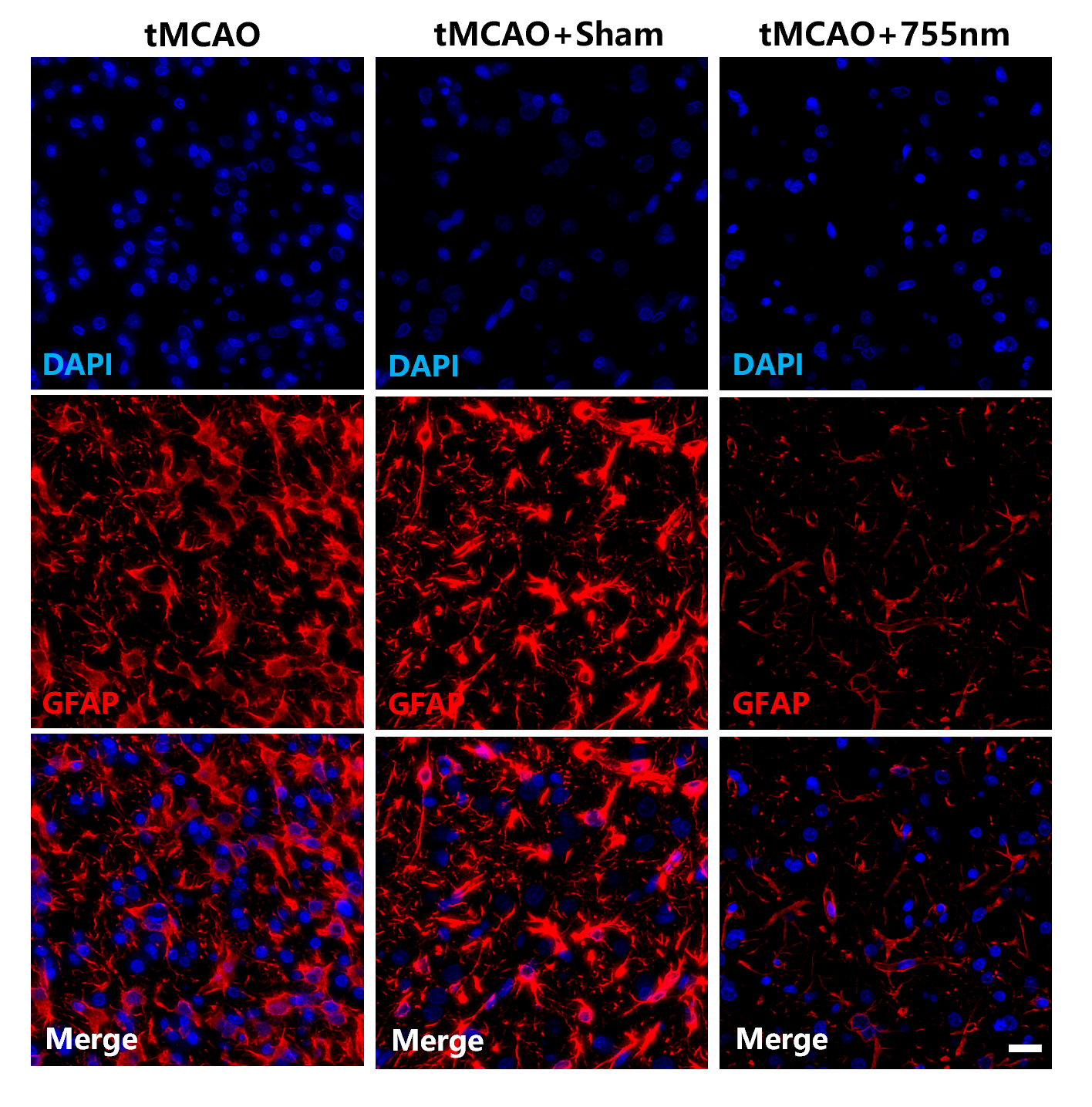


**Fig. S5.** Fluorescent images of astrocytes (GFAP) in the peri-infarct area 7 days after tMCAO. GFAP+ (red) and DAPI (blue), scale bar = 20 μm;

**STATISTICAL RESULTS TABLES**

Table S2. Statistical results of body weight in tMCAO rats in each group

| Body weight (g) | D0 | D1 | D3 | D7 | D14 | D21 | D28 |
| --- | --- | --- | --- | --- | --- | --- | --- |
| tMCAO | 321.3±3.616 | 283.8±5.073 | 261.3±6.327 | 245.2±9.574 | 260.7±13.97 | 295.7±13.74 | 318.7±13.78 |
| tMCAO+Sham | 309.6±4.145 | 278.9±8.011 | 266.3±9.063 | 250.9±10.21 | 254.2±10.82 | 277.0±12.12 | 307.3±12.73 |
| tMCAO+638 nm | 307.4±3.989 | 283.4±5.154 | 274.4±9.470 | 279.6±9.916 | 306.4±10.30 | 345.4±11.98 | 373.5±9.458 |
| tMCAO+755 nm | 312.3±3.547 | 299.2±3.524 | 288.8±6.937 | 292.7±6.827 | 326.8±10.08 | 364.0±9.544 | 382.9±9.862 |
| tMCAO+808 nm | 308.8±2.599 | 292.0±3.257 | 284.3±4.837 | 292.2±4.926 | 329.2±9.136 | 358.2±7.683 | 380.0±6.549 |
| *P1* | 0.1710 | 0.9633 | 0.9895 | 0.9893 | 0.9936 | 0.7657 | 0.9446 |
| *P2* | 0.0685 | >0.9999 | 0.7338 | 0.0491* | 0.0390* | 0.0239* | 0.0068** |
| *P3* | 0.4112 | 0.2569 | 0.0893 | 0.0026** | 0.0009*** | 0.0008*** | 0.0011** |
| *P4* | 0.1274 | 0.8102 | 0.2131 | 0.0029** | 0.0006*** | 0.0025** | 0.0020** |
| *P5* | 0.9926 | 0.9725 | 0.9409 | 0.1411 | 0.0133* | 0.0008*** | 0.0007*** |
| *P6* | 0.9847 | 0.0668 | 0.2300 | 0.0102* | 0.0003*** | <0.0001**** | 0.0001*** |
| *P7* | 0.9999 | 0.4127 | 0.4504 | 0.0114* | 0.0002*** | <0.0001**** | 0.0002*** |

All data are expressed as mean ± s.e.m., n = 10. Statistical significance was defined as *P* < 0.05, **P* < 0.05，***P* < 0.01，****P* < 0.001 or *****P* < 0.0001. *P1:* tMCAO vs. tMCAO+Sham, *P2*: tMCAO vs. tMCAO+638 nm, *P3:* tMCAO vs. tMCAO+755 nm, *P4*: tMCAO vs. tMCAO+808 nm, *P5*: tMCAO+Sham vs. tMCAO+638 nm, *P6*: tMCAO+Sham vs. tMCAO+755 nm, *P7*: tMCAO+Sham vs. tMCAO+808 nm.

Table S3. Statistical results of mNSS in tMCAO rats in each group

| mNSS | D1 | D3 | D7 | D14 | D21 | D28 |
| --- | --- | --- | --- | --- | --- | --- |
| tMCAO | 10.30±0.3667 | 8.700±0.5175 | 7.100±0.3480 | 5.800±0.3266 | 4.900±0.4819 | 3.700±0.4485 |
| tMCAO+Sham | 9.900±0.4069 | 8.900±0.6046 | 7.100±0.3145 | 6.300±0.4726 | 4.600±0.4522 | 3.100±0.2333 |
| tMCAO+638 nm | 9.900±0.4069 | 8.000±0.4472 | 5.500±0.4773 | 3.600±0.3055 | 2.600±0.2667 | 1.300±0.3000 |
| tMCAO+755 nm | 10.00±0.4216 | 8.200±0.4163 | 4.800±0.2494 | 3.700±0.2134 | 2.600±0.1633 | 1.100±0.3145 |
| tMCAO+808 nm | 8.100±0.3145 | 6.200±0.3590 | 5.000±0.2981 | 3.400±0.2211 | 1.900±0.2333 | 1.000±0.2108 |
| *P1* | 0.9473 | >0.9999 | >0.9999 | >0.9999 | >0.9999 | >0.9999 |
| *P2* | 0.9473 | >0.9999 | 0.1706 | 0.0125* | 0.0399* | 0.0080** |
| *P3* | 0.9813 | >0.9999 | 0.0031** | 0.0220* | 0.0361* | 0.0020** |
| *P4* | 0.0019** | 0.0124* | 0.0104* | 0.0038** | 0.0002*** | 0.0010** |
| *P5* | >0.9999 | >0.9999 | 0.1634 | 0.0041** | 0.0636 | 0.0386* |
| *P6* | 0.9997 | >0.9999 | 0.0029** | 0.0076** | 0.0578 | 0.01128* |
| *P7* | 0.0153* | 0.0049** | 0.0098** | 0.0011** | 0.0004*** | 0.0062** |

All data are expressed as mean ± s.e.m., n = 10. Statistical significance was defined as *P* < 0.05, **P* < 0.05，***P* < 0.01，****P* < 0.001 or *****P* < 0.0001. *P1:* tMCAO vs. tMCAO+Sham, *P2*: tMCAO vs. tMCAO+638 nm, *P3:* tMCAO vs. tMCAO+755 nm, *P4*: tMCAO vs. tMCAO+808 nm, *P5*: tMCAO+Sham vs. tMCAO+638 nm, *P6*: tMCAO+Sham vs. tMCAO+755 nm, *P7*: tMCAO+Sham vs. tMCAO+808 nm.

Table S4. Statistical results of BSV based on T2WI in tMCAO rats in each group

| BSV | D1 | D7 | D14 | D21 | D28 |
| --- | --- | --- | --- | --- | --- |
| tMCAO组 | (117.0±2.252)% | (101.3±0.907)% | (95.85±1.227)% | (91.97±0.512)% | (92.34±0.630)% |
| tMCAO+Sham组 | (118.5±1.020)% | (102.6±0.837)% | (94.74±1.542)% | (93.18±1.091)% | (92.18±1.237)% |
| tMCAO+638 nm组 | (115.8±1.783)% | (101.3±1.088)% | (97.11±1.725)% | (95.26±2.318)% | (92.11±1.191)% |
| tMCAO+755 nm组 | (115.6±1.731)% | (101.4±0.922)% | (98.01±1.352)% | (94.36±1.139)% | (95.13±0.600)% |
| tMCAO+808 nm组 | (110.6±2.149)% | (100.8±1.126)% | (96.41±1.022)% | (96.95±0.864)% | (95.38±1.275)% |
| *P1* | 0.9790 | >0.9999 | 0.9789 | 0.9672 | >0.9999 |
| *P2* | 0.9900 | 0.9881 | 0.9679 | 0.4269 | 0.9998 |
| *P3* | 0.9823 | 0.9994 | 0.8076 | 0.7124 | 0.3365 |
| *P4* | 0.1265 | 0.9990 | 0.9985 | 0.0929 | 0.2585 |
| *P5* | 0.8403 | 0.9968 | 0.7515 | 0.8020 | >0.9999 |
| *P6* | 0.8031 | >0.9999 | 0.4754 | 0.9688 | 0.2841 |
| *P7* | 0.0391* | 0.9945 | 0.9130 | 0.2936 | 0.2149 |

All data are expressed as mean ± s.e.m., n = 6. Statistical significance was defined as *P* < 0.05, **P* < 0.05，***P* < 0.01，****P* < 0.001 or *****P* < 0.0001. *P1:* tMCAO vs. tMCAO+Sham, *P2*: tMCAO vs. tMCAO+638 nm, *P3:* tMCAO vs. tMCAO+755 nm, *P4*: tMCAO vs. tMCAO+808 nm, *P5*: tMCAO+Sham vs. tMCAO+638 nm, *P6*: tMCAO+Sham vs. tMCAO+755 nm, *P7*: tMCAO+Sham vs. tMCAO+808 nm.

Table S5. Statistical results of BLV based on T2WI in tMCAO rats in each group

| BLV | D1 | D7 | D14 | D28 |
| --- | --- | --- | --- | --- |
| tMCAO | (23.40±0.939)% | (23.77±3.298)% | (29.27±2.395)% | (29.20±1.359)% |
| tMCAO+Sham | (22.43±2.929)% | (22.65±1.975)% | (28.88±2.149)% | (31.68±2.025)% |
| tMCAO+638 nm | (21.03±1.326)% | (14.84±0.663)% | (21.18±1.331)% | (20.95±0.887)% |
| tMCAO+755 nm | (22.29±1.576)% | (14.73±1.468)% | (17.75±1.348)% | (19.24±1.474)% |
| tMCAO+808 nm | (13.04±4.091)% | (9.997±1.820)% | (18.46±4.294)% | (14.14±1.436)% |
| *P1* | 0.9986 | 0.9949 | >0.9999 | 0.7607 |
| *P2* | 0.9593 | 0.0346* | 0.1961 | 0.0048** |
| *P3* | 0.9976 | 0.0317* | 0.0278* | 0.0006*** |
| *P4* | 0.0466* | 0.0006*** | 0.0431* | <0.0001**** |
| *P5* | 0.9942 | 0.0797 | 0.2355 | 0.0002*** |
| *P6* | >0.9999 | 0.0737 | 0.0354* | <0.0001**** |
| *P7* | 0.0837 | 0.0015** | 0.0544 | <0.0001**** |

All data are expressed as mean ± s.e.m., n = 6. Statistical significance was defined as *P* < 0.05, **P* < 0.05，***P* < 0.01，****P* < 0.001 or *****P* < 0.0001. *P1:* tMCAO vs. tMCAO+Sham, *P2*: tMCAO vs. tMCAO+638 nm, *P3:* tMCAO vs. tMCAO+755 nm, *P4*: tMCAO vs. tMCAO+808 nm, *P5*: tMCAO+Sham vs. tMCAO+638 nm, *P6*: tMCAO+Sham vs. tMCAO+755 nm, *P7*: tMCAO+Sham vs. tMCAO+808 nm.

Table S6. Effects of 755 nm laser on the expression and morphology of microglia markers in tMCAO rats

| 组别 | Iba-1 Mean Intensity (A.U.) | No. of branches/cell | No. of teriminal processes/cell | Branch length/cell (mm) |
| --- | --- | --- | --- | --- |
| tMCAO | 97.50±8.389 | 20.37±0.9610 | 8.546±0.2909 | 8.920±0.1217 |
| tMCAO+Sham | 87.70±10.77 | 20.07±0.6383 | 8.419±0.3000 | 8.833±0.2112 |
| tMCAO+755 nm | 55.54±6.826 | 24.92±1.344 | 10.23±0.4515 | 9.915±0.1101 |

All data are expressed as mean ± s.e.m., n = 3-4.

Table S7. Statistical results of OFT and NOR tests

|  | Total distance traveled (cm) | Move time (s) | Center movement time (s) | Discrimination Index |
| --- | --- | --- | --- | --- |
| Normal | 5257±326.2 | 478.2±9.049 | 32.42±5.722 | 0.7180±0.03428 |
| tMCAO | 4217±441.1 | 348.3±43.90 | 9.470±2.258 | 0.3229±0.04629 |
| tMCAO+Sham | 4028±453.0 | 390.2±27.07 | 7.953±1.699 | 0.3708±0.05846 |
| tMCAO+755 nm | 4698±702.2 | 370.5±40.29 | 26.14±4.632 | 0.6396±0.04064 |

All data are expressed as mean ± s.e.m., n = 10-14.

Table S8. Statistical results of MWM tests training period

|  | D1 | D2 | D3 | D4 | D5 |
| --- | --- | --- | --- | --- | --- |
| Normal | 29.99±1.942 | 20.32±1.395 | 14.18±1.351 | 12.17±0.9444 | 9.827±0.4671 |
| tMCAO | 53.59±3.622 | 40.36±5.719 | 33.02±6.270 | 29.14±8.841 | 21.97±3.604 |
| tMCAO+Sham | 54.29±2.384 | 43.45±4.334 | 35.66±6.725 | 26.70±7.091 | 28.12±5.448 |
| tMCAO+755 nm | 44.26±3.966 | 24.01±5.854 | 21.91±5.905 | 6.320±0.8660 | 8.670±1.294 |

All data are expressed as mean ± s.e.m., n = 6-8.

Table S9. Statistical results of MWM tests without platform

|  | Swimming speed (cm/s) | Distance in target quadrant (cm) | Time in target quadrant (s) |
| --- | --- | --- | --- |
| Normal | 28.62±1.857 | 698.5±53.15 | 24.80±1.160 |
| tMCAO | 23.44±1.457 | 265.3±21.94 | 15.13±1.955 |
| tMCAO+Sham | 23.70±1.196 | 324.0±34.06 | 16.67±1.902 |
| tMCAO+755 nm | 24.62±1.016 | 497.4±17.49 | 24.09±0.6573 |

All data are expressed as mean ± s.e.m., n = 6-8.

Table S10. ECS structure and ISF drainage parameters in tMCAO rats in each group rats based on Tracer-based MRI of stereotactic injection

| 组别 | T_1/2_ (min) | D*(×10^-4^mm^2^/s) | α(%) | λ |
| --- | --- | --- | --- | --- |
| Normal | 63.98±5.375 | 4.277±0.1184 | 18.25±0.1198 | 1.577±0.02182 |
| tMCAO | 141.8±12.07 | 3.468±0.1768 | 17.51±0.2270 | 1.757±0.04732 |
| tMCAO+Sham | 139.6±13.68 | 3.600±0.09563 | 17.47±0.1032 | 1.718±0.02313 |
| tMCAO+755 nm | 79.72±3.689 | 4.284±0.1019 | 18.19±0.1044 | 1.575±0.01867 |

All data are expressed as mean ± s.e.m., n = 6. T_1/2_: half-life, D*: Diffusion rate, α: volume fraction, λ: tortuosity.
