# Supplementary figures and images for: Accelerated Molecular Transportation in the Brain Extracellular Space with 755-nm Light Attenuates Post-Stroke Cognitive Impairment in Rats
Source: Cyborg Bionic Syst. 2025 May 6;6:0262. doi: 10.34133/cbsystems.0262 (PMC12053100; doi:10.34133/cbsystems.0262)

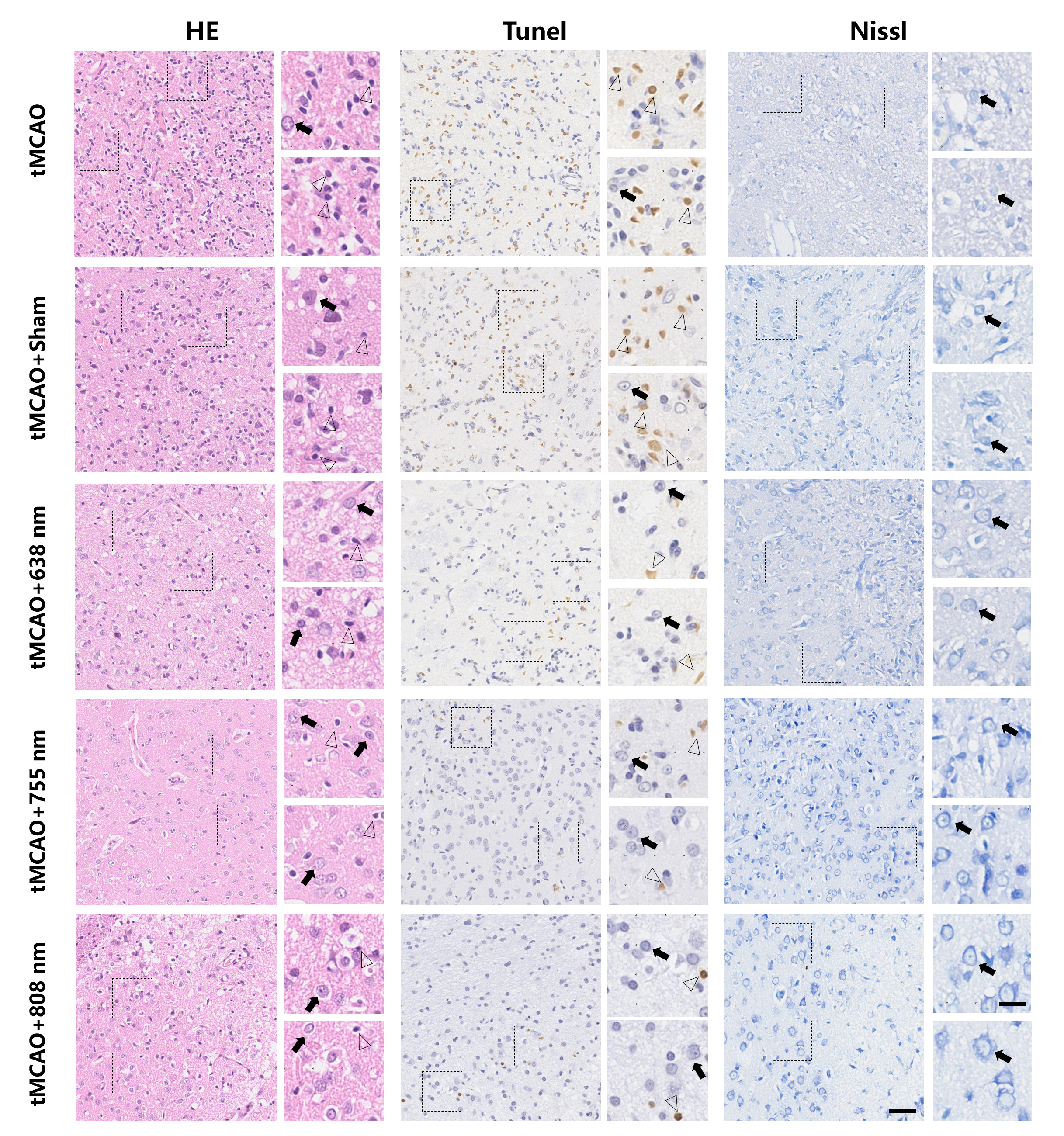

Supplement: Supplementary 1 — Materials and Methods Figs. S1 to S5 Tables S1 to S10 [file cbsystems.0262.f1.zip › S1.tif]

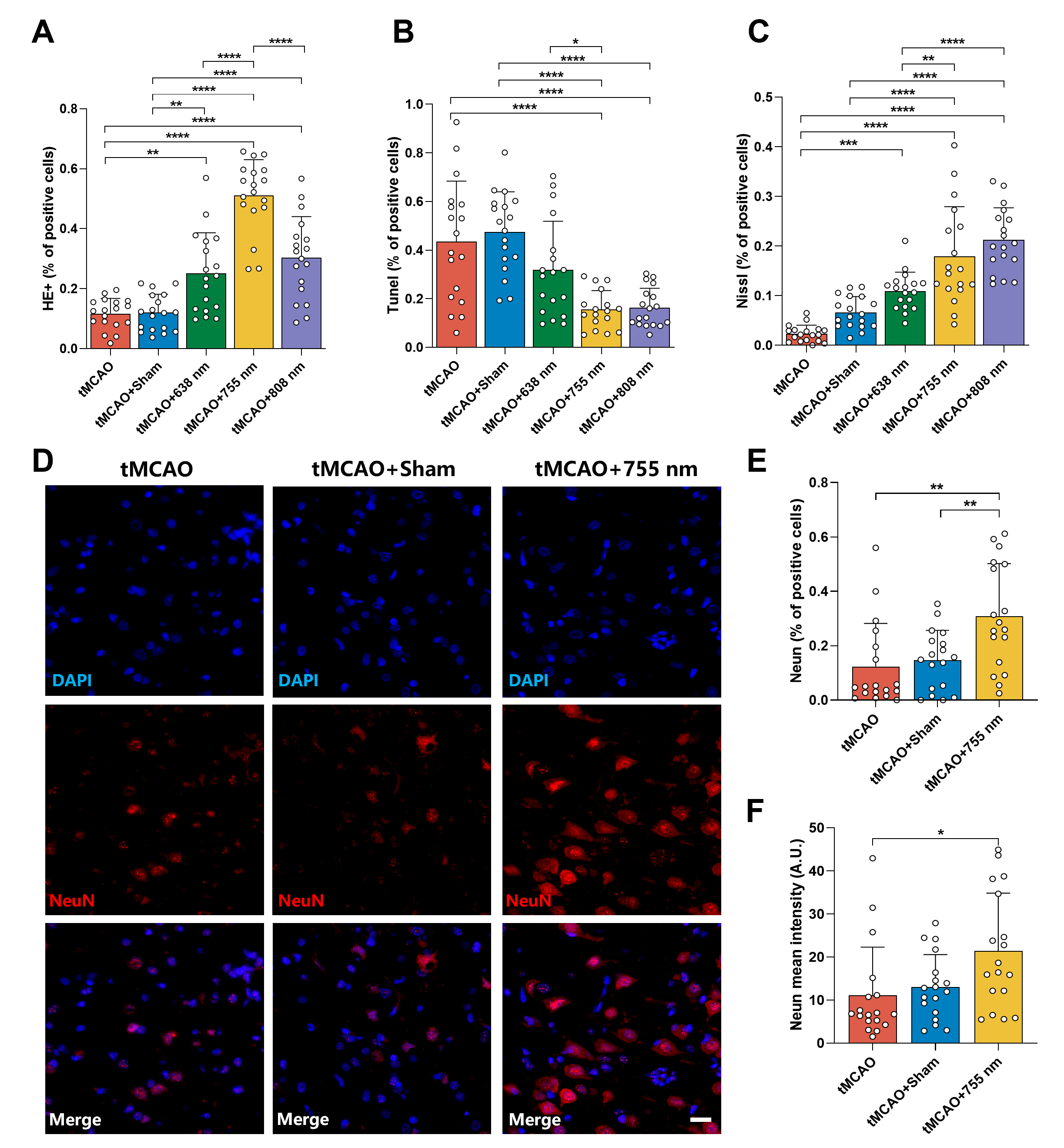

Supplement: Supplementary 1 — Materials and Methods Figs. S1 to S5 Tables S1 to S10 [file cbsystems.0262.f1.zip › S2.tif]

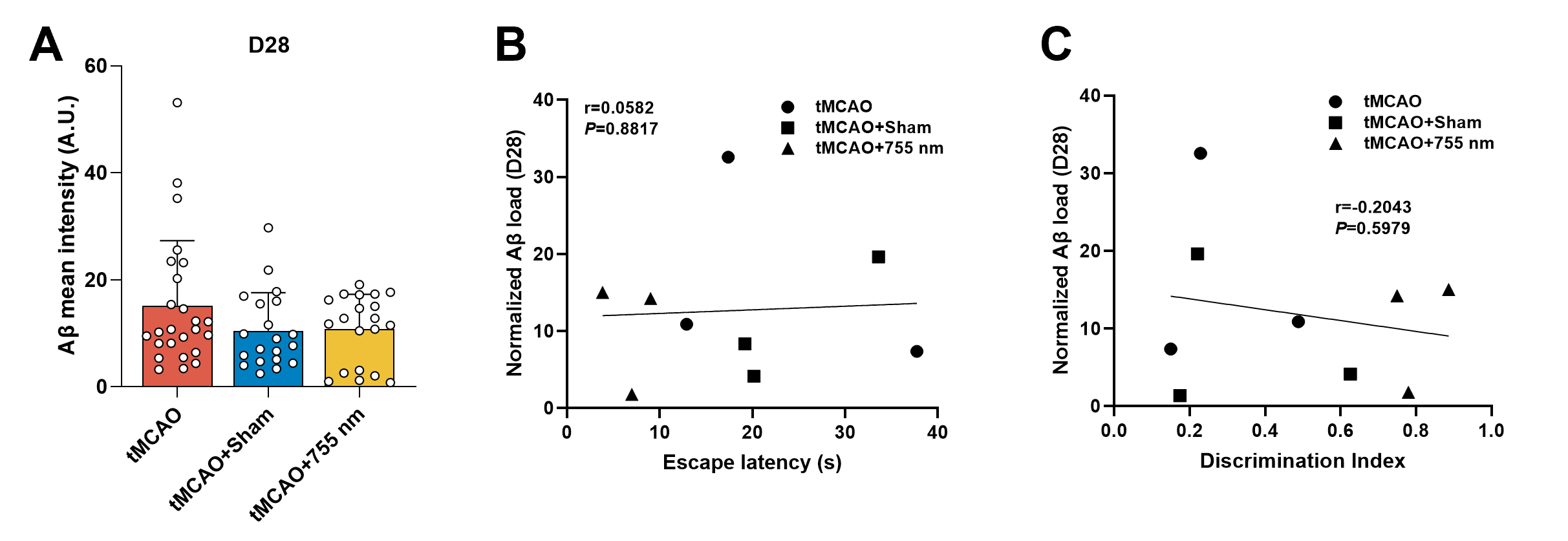

Supplement: Supplementary 1 — Materials and Methods Figs. S1 to S5 Tables S1 to S10 [file cbsystems.0262.f1.zip › S3.tif]

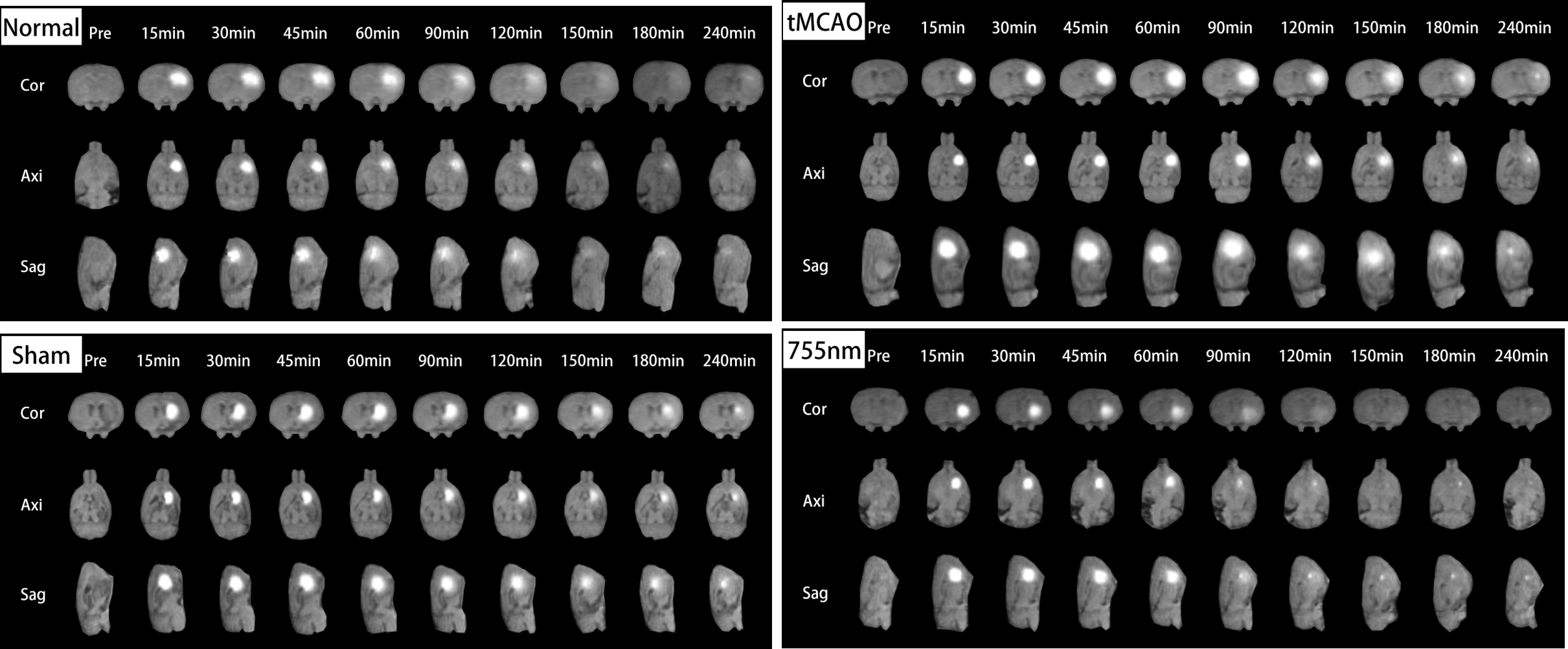

Supplement: Supplementary 1 — Materials and Methods Figs. S1 to S5 Tables S1 to S10 [file cbsystems.0262.f1.zip › S4.tif]

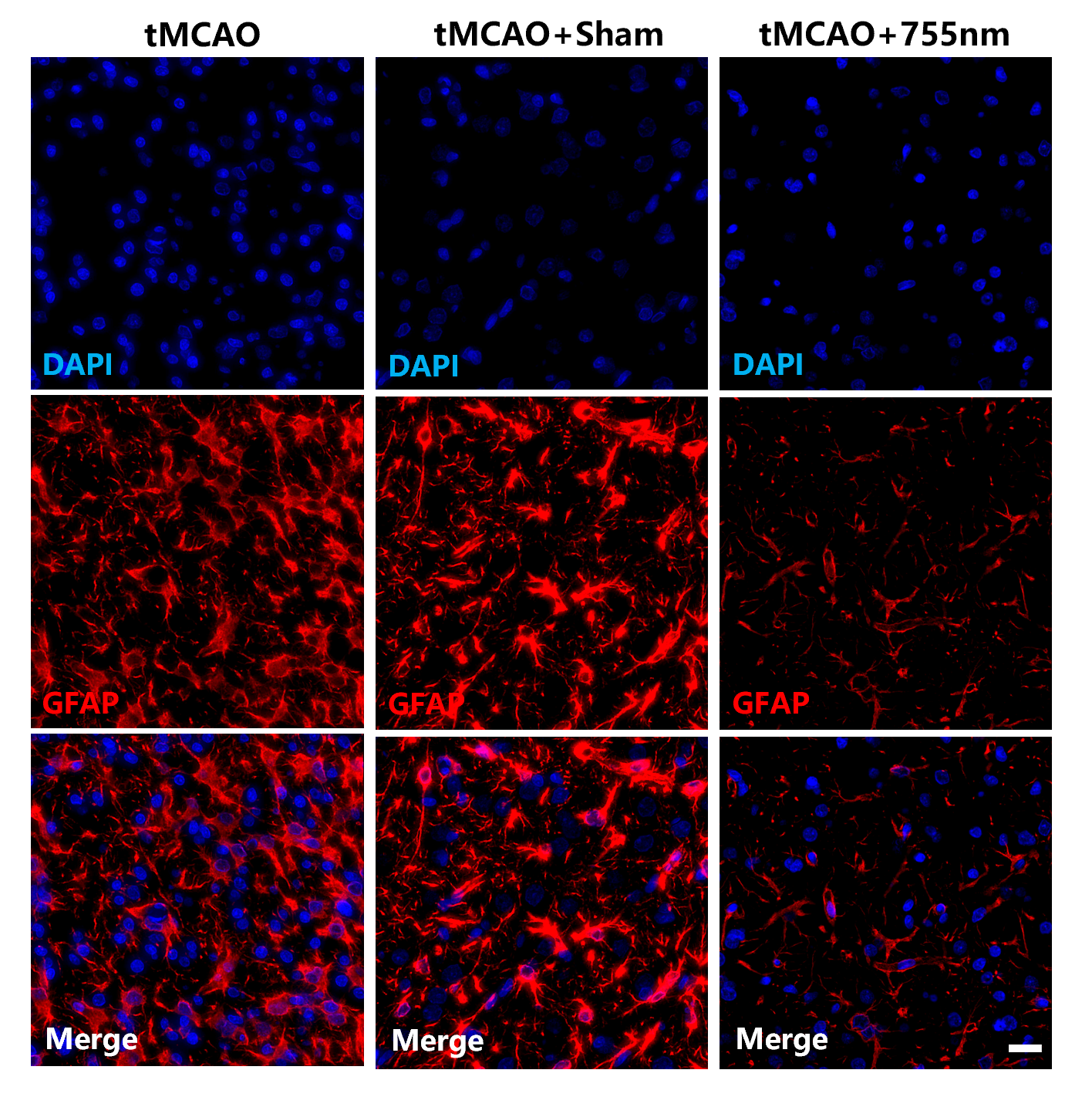

Supplement: Supplementary 1 — Materials and Methods Figs. S1 to S5 Tables S1 to S10 [file cbsystems.0262.f1.zip › S5.tif]
